# Patch depletion, niche structuring and the evolution of co-operative foraging

**DOI:** 10.1186/1471-2148-11-335

**Published:** 2011-11-17

**Authors:** Daniel J van der Post, Dirk Semmann

**Affiliations:** 1 Courant Research Centre Evolution of Social Behaviour, Georg-August Universität Göttigen, Kellnerweg 6, 37077, Göttingen, Germany; 2 Institute of Artificial Intelligence, University of Groningen, P.O. Box 407, 9700 AK, Groningen, The Netherlands; 3 Behavioural Ecology and Self-Organization, University of Groningen, PO Box 11103, 9700 CC Groningen, The Netherlands

## Abstract

**Background:**

Many animals live in groups. One proposed reason is that grouping allows cooperative food finding. Group foraging models suggest that grouping could increase food finding rates, but that such group processes could be evolutionarily unstable. These models assume discrete food patches which are fully detectable. However, often animals may only be able to perceive local parts of larger-scale environmental patterns. We therefore use a spatial individual-based model where food patches are aggregates of food items beyond the scale of individual perception. We then study the evolution of foraging and grouping behavior in environments with different resource distributions.

**Results:**

Our results show that grouping can evolve to increase food intake rates. Two kinds of grouping evolve: traveling pairs and opportunistic grouping, where individuals only aggregate when feeding. Grouping evolves because it allows individuals to better sense and deplete patches. Such enhanced patch depletion is particularly apparent on fragmented and partially depleted patches, which are especially difficult for solitary foragers to deplete. Solitary foragers often leave a patch prematurely because a whole patch cannot be observed directly. In groups, individuals that are still eating allow other individuals that inadvertently leave the patch, to return and continue feeding. For this information sharing a grouping tendency is sufficient and observing whether a neighbor is eating is not necessary. Grouping therefore leads to a release from individual sensing constraints and a shift in niche specialization, allowing individuals to better exploit partially depleted patches.

**Conclusions:**

The evolved group foraging can be seen as cooperative in the sense that it leads to a mutually-beneficial synergy: together individuals can achieve more than on their own. This cooperation exists as a group-level process generated by the interaction between grouping and the environment. Thus we reveal how such a synergy can originate in evolution as a side-effect of grouping via multi-level selection. Here there is no cooperative dilemma as individuals cannot avoid producing information for their neighbors. This scenario may be a useful starting point for studying the evolution of further social and cooperative complexity.

## Background

Why many animals live and forage in groups has long been a topic of interest [1,2]. Historically, the two main explanations for the evolution of group foraging are (i) increased food finding efficiency and (ii) a response to predation, especially in mammals and birds [2]. The present evidence, based on information sharing (IS) and producer scrounger (PS) models [3-10], both provide support for and against increased food finding efficiency due to grouping, thereby bringing its relevance for the evolution of grouping into question [2].

For some information sharing models it is assumed that individuals find food patches, neighbors join them on those patches, and that all individuals attain an equal share of patch contents [4,5,11]. Here the per capita patch finding rates in groups are assumed to be equal to those of solitary foragers. Results from these models predict that the decrease in the proportion of a patch eaten per capita due to grouping, cancels out any gains in patch finding success due to grouping [4,6,10]. However, if patches are highly ephemeral and rare, or individuals leave patches after becoming satiated (and don't return), grouping can be shown to increase food finding rates [5,6,11].

One assumption in information sharing models is that searching for food and monitoring whether neighbors have found food is fully compatible (an idealization) [4-6,10]. This allows individuals to instantaneously benefit from patches detected by others, while not foregoing any opportunities to join their neighbors while searching for their own food. However, if searching and monitoring are incompatible, as assumed in producer-scrounger (PS) models [8,12,13], then foraging groups can suffer from information parasitism [8,12,13]. Scroungers do not search and produce information, because scroungers only monitor neighbors and take advantage of the search effort of others (producers). In IS models this cooperative dilemma is ignored. PS models suggest that scrounging will inevitably evolve in foraging groups, which will reduce the overall patch finding rate of groups [8], and makes that it is unlikely that group foraging can lead to greater food finding rates. Instead, it has been suggested that because grouping can reduce the variance of food intake, group foraging could be a risk-averse foraging strategy [3,4,6,7,10].

Another assumption in IS models, is that the per capita patch finding rates in groups is equal to those of solitary foragers, i.e. patch finding rates in groups scale linearly with group size [4-6]. However, the convergence in space of individuals due to grouping likely increases overlap in areas searched, reducing per capita patch finding rates in groups [10]. Moreover, various factors such as reduced information sharing in groups, interference, and competition, could further reduce food finding efficiency in groups [6,8-10]. Indeed, a more recent study already directly assumes reduced patch finding efficiency in groups [14]. 

In apparent contradiction to the assumption that group foraging will lead to reduced patch finding rates, an interesting simulation study shows that group foraging can evolve in patchy environments [15]. Wood and Ackland [15] use a spatial agent-based model where grouping evolves through inherited changes in local individual interactions that lead to self-organized grouping. In their model, patches are aggregations of food items, and such modeled environments are referred to as "continuous" patchy environments [16,17]. Patches therefore exist, and can be detected, only when there are food items aggregated at a certain location. In such environments, staying in a patch in order to deplete it, is difficult, especially if food items can only be detected very locally. Shore birds, for example, often probe for underground prey and face obvious visibility constraints when foraging [18]. Therefore, food patches cannot be directly observed and individuals can very easily leave a patch without realizing it. In optimal search theory (OST), strategies have been proposed where individuals conduct intensive searching upon finding a food item, and switch to extensive search after not finding food for a while [17]. Such a strategy allows search to become concentrated in the right areas and allows a patch to be detected, by so called area-concentrated search [17]. In contrast, in IS and PS theory, patches are assumed to be discrete and once detected can be fully depleted ("discrete" patchy environments).

If patches (or larger-scale patterns of resources) are difficult to detect and deplete, the assumptions made in IS and PS models, about how individuals share information and patch contents, are unlikely to hold: it becomes unclear what information individuals and groups have access to, and what proportion of a patch individuals can deplete. Instead, if patchiness exists on a scale that is beyond the local perception of individuals, then grouping may play a role in cooperatively sensing, detecting and depleting patches. Various non-evolutionary simulation studies have shown that aggregates of individuals can collectively sense the environment on a scale that exceeds individual-level perception, from photo- and thermo-taxis in Dictyostelium amoebae [19,20], groups sensing concentration gradients [21], to cumulative cultural diet improvement in group foragers [22]. In Wood and Ackland's model such processes could also be at work [15].

Here we study the evolution of grouping tendencies of foragers in environments varying in patchiness and ask: can group foraging evolve in environments where there are patterns of resource availability that extend beyond the local perception of individuals? Like Wood and Ackland [15], grouping is not directly predefined, but evolution of grouping parameters could lead to different kinds of grouping through self-organization. Compared to Wood and Ackland, we impose a stronger local constraint on food detection range, to specifically focus on how local information processing can become scaled up through grouping. Moreover, we study overlapping generations, so that we can study evolution in environments with existing population and ecological structure. Our population is not fixed in size, but grows relative to replenishing resources, which means that Darwinian fitness is always relative and emerges from competition over finite resources. From an evolutionary point of view, this means that we study the origin of novel social phenomena and their side-effects. This is possible because predefinitions are only made on an individual-level, while group-level processes can emerge via self-organization. These group-level processes can then potentially generate novel selection pressures. We then study if, how and why grouping evolves, specifically focusing on patch detection and depletion, types of grouping, and foraging efficiency. We consider this study as a baseline for models with more elaborated individual-level behavior and more detailed environmental structure.

Using this model we show that grouping evolves to increase foraging efficiency by generating group-level sensing of food patches in order to "stay in a patch" to better deplete it. Group foraging leads to a change in niche specialization, which is what allows grouping to generate enhanced foraging efficiency.

## Methods

We use an individual-based model which consists of (i) an environment with food, (ii) foraging behavior, (iii) grouping behavior and (iv) reproduction and mutation. This extends our model on solitary foraging [23] with grouping behavior. Below we give a model summary which is sufficient to understand our results (for further model details see Table 1 and Section 1 in Additional file 1). We organize our description in a "spatio-temporal scaling section" which sets the context in which we evolve behavior, described in an "evolvable behavior" section.

**Table 1:** Non-evolvable model specifications defining the scaling context

Category	Parameter/description	Value	Units
Time scale	*t*_*MIN *_(minimal action duration)	10	sec
	day (daylight hours)	720*6	*t*_*MIN*_
	*(year)	365	day
Spatial scale	individual movement	continuous	
	resources placement grid	1	m
Local information processing	*t*_*E *_(handling time)	1	*t*_*MIN*_
	resource detectability range	2	m
	*d*_*R *_(individual reach)	0.9	m
	*r*_*D *_(resource detectability)	0.1	per *t*_*MIN *_per *m*^2^
	*z*_*M *_(max neighbor awareness)	50	m
	*z*_*L *_(alignment zone)	25	m
	*(minimal action duration)	1	*t*_*MIN*_
	*(maximum speed)	1	m/*t*_*MIN*_
Energy and life-history	*E*_*r *_(energy per item)	2	units
	*E*_*m *_(metabolism)	1/6	units/*t*_*MIN*_
	*E*_*M *_(maximum energy)	100000	units
	minimal energy	0	units
	requirement to give birth	*E*_*M*_	units
	birth energy costs	*E*_*M*_/2	units
	offspring energy	*E*_*M*_/2	units
	death rate	0.1	per year
	maximum age	10	years
	mutation rate	0.05	
	*(*t*_*MIN_EAT *_(minimal eat in-terval: search and eat))	*t*_*MIN *_+ *t*_*E*_	*t*_*MIN*_
	*(*t*_*MAX*_*EAT *_(to compensate for metabolism))	*E*_*r*_/*E*_*m*_	*t*_*MIN*_
	*(max net energy gain)	(*E*_*r*_/*t*_*MIN_EAT*_) - *E*_*m*_	units/*t*_*MIN*_
	*(minimal birth interval)	*E*_*M*_/(2((*E*_*r*_/*t*_*MIN_EAT*_) - *E*_*m*_))	*t*_*MIN*_
Environment	*R*_*g *_(resource influx rate)	1.1	items/*t*_*MIN*_
	*R*_*r *_(resource renewal interval)	1	year
	*A *(field size)	5660 × 5660	m
	*(carrying capacity)	*R*_*g*_*t*_*MAX_EAT*_	inds
	*(*R*_*T *_(total food items))	*R*_*g*_*R*_*r*_	items
	*(average resource density)	*R*_*T*_/*A*	items per *m*^2^

Specifications designated by *() derive from explicitly chosen parameter values, giving 23 direct specifications and 10 derived specifications (non-independent).

### Spatio-temporal scaling

#### Environment

The environment is a 2-dimensional 5660 by 5660 meter continuous space (about 32 *km*^2^), which is large enough to implement a variety of resource distributions and supports a viably evolving population of about 120 individuals (where our simulations are still fast enough). Each year is 365 days, and for each day we consider "daylight" (720 minutes). Individuals and resources can be positioned at any given location. For convenience we place resource items on intersections of a 1 by 1 meter lattice (as in [24]). We keep the total amount of food constant and only vary the distribution of resources, with the following classes of patchiness: (I) uniform (no patches), (II) low (19653 patches, 40 meter diameter, 1 resource item/*m*^2^), (III) medium (8000 patches, 40 m diameter, 2 resource items/*m*^2^) (IV) high (5333 patches, 40 m diameter, 3 resource items/*m*^2^), (V) big patches (2000 patches, 80 m diameter, 2 resource items/*m*^2^). Patches are randomly placed in the environment, and all food items of a patch appear at the same time each year. Appearance times of patches vary randomly throughout a year. Food items are depleted once eaten, and remain depleted until that patch re-appears the next year. (For the uniform environment the same is true, but then on a food item level). We run simulations for 1000 years which is 2,628,000,000 time steps (each 10 seconds), where about 120 individuals make decisions and execute behavioral actions.

#### Local information processing

Food can be detected up to 2 meters and individuals must therefore move around to find food. Individuals cannot directly observe larger scale patterns of resources. The minimal time interval (10 seconds), defines a maximum movement speed of 360 meters per hour. The maximal distance at which individuals can detect each other (50 meters) is such that the world is big enough to make grouping difficult (i.e. individuals can lose each other and be out of view) and allows a clear distinction between solitary and grouping behavior. This range is not unreasonable for many foragers such as primates. Individuals have a set of behavioral actions that take time, and are sequential. The individual which is next to execute an action is the one with the shortest time left to complete an action.

#### Reproduction and Population

Individuals need to find food to gain energy, and their birth interval (reproductive rate) depends on energy intake rate. Energy per food item, energy metabolism, and energy to give birth are such that the inter-birth interval is in the order of months to years. Individuals can live for maximally 10 years, which covers several reproductive events. Patches are depleted in the order of hours-days, so individuals need to visit many patches within a reproductive cycle. Reproductive success is therefore dependent on how individuals react to the full scale of environmental patterns. The forager population grows until resource depletion reaches the point where births replace deaths (carrying capacity). Competition for resources is therefore generated.

#### "Genes", mutation and natural selection

The parameters defining decision making, behavioral actions and grouping tendencies (see descriptions below and Table 2) are inherited as "genes" by offspring, with some mutational error (0.05 probability per gene). This genetic variation and variation in foraging success (birth intervals), in combination with competition for resources, generates natural selection.

The complete set of model specifications defining the scaling context are given in Table 1. Note that various higher-level specifications are defined in terms of smaller-scale specifications, reducing the overall dimensionality (see Section 1.1. in Additional file 1 for more detailed discussion on this issue). We expect parameter combinations that satisfy the qualitative relationships of these parameters to give similar results. Nonetheless, the parameter space is too large to address here, and like any model study we accept that there is a set of invariant assumptions (whether implicit or explicit) that set the context in which our analysis takes place (see Section 1.5 in Additional file 1 for more discussion on the issue of parameters and assumptions).

**Table 2:** Evolvable parameters of individuals

Category	Parameter	St. dev.	Min	Max	Units
**Grouping**					
	*n*_*R *_(tolerated neighbors)	0.2(15)	0.0	-	individuals
Zones	*z*_*R*_	0.2(15)	0	-	m
	*z*_*A*_	0.2(15)	*z*_*R*_	-	m
Angles	*a*_*R*_	0.2(360)	0.0	360	degrees
	*a*_*A*_	0.2(360)	0.0	360	degrees

**Foraging**					
Durations	*t*_*M *_(MOVE)	0.2(2)	1	18	*t*_*MIN*_
	*t*_*F *_(FOODSCAN)	0.2(2)	1	18	*t*_*MIN*_
Distances	*d*_*M *_(MOVE)	0.2(15)	0.0	-	m
	*d*_*F *_(FOODSCAN)	0.2(15)	0.0	-	m
Angles	*a*_*M *_(MOVE)	0.2(360)	0.0	360	degrees
	*a*_*F *_(FOODSCAN)	0.2(360)	0.0	360	degrees
Probabilities	*p*_*M *_(MOVE after MOVE)	0.2	0.0	-	
	*p*_*SE *_(FOODSCAN after EAT)	0.2	0.0	-	
	*p*_*SN *_(FOODSCAN after NOFOOD)	0.2	0.0	-	
	*p*_*MTF *_(MOVETOFOOD)	0.2	0.0	-	

The value of mutated genes is drawn from a normal distribution about the mother's gene value. The standard deviation of this normal distribution is scaled (0.2(x)) relative to what is considered a reasonable range for the parameter. The maximum of durations is imposed due to how the model was programmed, but is high enough not to affect the results. *t*_*MIN *_is the minimal time interval (see Section 1.1. in Additional file 1 ).

### Evolvable behavior

#### Foraging

**Figure 1:**
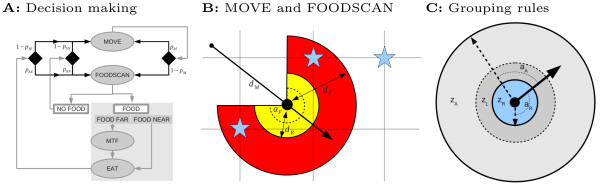
**Foraging and grouping**. (A) Decision making. Ovals: behavior actions (MTF = move to food); Squares: information acquired from the environment; Diamonds: decision points. Arrows indicate the sequence of actions, decision points, and information. Grey arrows: fixed; Black arrows: evolvable probabilities, namely *p*_*M *_(repeat MOVE), *p*_*SN *_(FOODSCAN after not finding food), *p*_*SE *_(FOODSCAN after eating). The shaded gray square indicates fixed behavior that occurs in the "FOOD" context. (B) Visual representation of foraging: *d*_*M *_is the distance covered with MOVE (solid line), followed by a FOODSCAN (red + yellow) of angle *a*_*F *_(dashed line) about forward direction (thickest arrow) over distance *d*_*F*_. Food items (blue stars) can then be detected. If food is beyond reach *d*_*R *_(yellow), then the individual will MOVETOFOOD (to the closest star detected) before EAT. (C) Grouping rules: repulsion zone (*z*_*R*_, inner, blue), alignment zone (*z*_*L*_, middle, dark gray), attraction zone (*z*_*A*_, outer, light gray). *z*_*R *_and *z*_*A *_are evolvable, and *z*_*L *_is fixed. Thick arrow: individual's heading; Shortest arrow: maximum turn when repulsed *a*_*R*_; Longest arrow: maximum turn when attracted and aligning *a*_*A*_.

To search for food, individuals have a simple decision making algorithm and a set of behavioral actions (MOVE, FOODSCAN, MOVETOFOOD and EAT), which can occur in a given sequence (Figure 1a-b). We initialize simulations with parameters evolved in solitary foragers [23], which cause individuals to alternate MOVE and FOODSCAN (i.e. individuals MOVE after not finding food) (see Table S2 in Additional file 1 for initialization values). If individuals find food, individuals EAT, or MOVETOFOOD if food is out of reach. After EAT, individuals do FOODSCAN again (i.e. until no more food is found at that location). For an overview of all evolvable parameters see Table 2. In our simulations, foraging parameters remained approximately on initial values, indicating that grouping did not significantly feed back on foraging parameters (see Section 2 in Additional file 1).

#### Grouping

During MOVE actions, individuals can change direction in relation to the position of neighbors, which allows grouping behavior. We base evolvable grouping rules on repulsion-alignment-attraction models which focus mainly on schools of fish and flock of birds [15,25-28]. We have adapted our model in terms of what we consider reasonable for group foragers such as primates. In our case grouping works as follows (Figure 1c):

(i) When there are more than *n*_*R *_neighbors in the repulsion zone *z*_*R*_, individuals turn away from those neighbors with maximal angle *a*_*R *_towards a preferred direction $\left({\vec{d}}_{i}\right)$ given by:

(1) $${\vec{d}}_{i}=-\frac{\sum_{j\neq i}^{n_{RZ}}{\vec{r}}_{ij}}{\left|\sum_{j\neq i}^{n_{RZ}}{\vec{r}}_{ij}\right|}$$

where ${\vec{r}}_{ij}=\left({\vec{p}}_{j}-{\vec{p}}_{i}\right)∕|\left({\vec{p}}_{j}-{\vec{p}}_{i}\right)|$ is the unit vector in the direction of neighbor *j*, while ${\vec{p}}_{i}$ is the position of individual *i*, and *n*_*RZ *_is the number of neighbors in the repulsion zone.

(ii) Otherwise (if *n*_*RZ *_<*n*_*R*_), individuals turn towards individuals in a larger attraction zone *z*_*A *_and the average direction of individuals in an alignment zone *z*_*L *_with maximal angle *a*_*A*_, where the preferred direction is:

(2) $${\vec{d}}_{i}=\frac{\sum_{j\neq i}^{n_{A}}{\vec{r}}_{ij}+\sum_{j\neq i}^{n_{L}}{\vec{v}}_{j}}{\left|\left(\sum_{j\neq i}^{n_{A}}{\vec{r}}_{ij}+\sum_{j=i}^{n_{L}}{\vec{v}}_{j}\right)\right|}$$

where *n*_*L *_is the number of individuals between the repulsion and alignment zone, *n*_*A *_is the number of neighbors between the repulsion zone and attraction zone, and ${\vec{v}}_{j}$ is the unit direction vector of neighbor *j*. If the preferred direction is less than the maximal turning angle (*a*_*R *_or *a*_*A*_), then the individual only turns up to its preferred direction.

We included the tolerated number of neighbors parameter, *n*_*R*_, to allow for greater regulation of group size. Thus we assume that forager grouping tendencies can be regulated by how many neighbors an individual has. Since we include "pause-travel" foraging behavior, where individuals stop to search for food and eat, we do not consider that movement speed restricts the angle with which individuals can change direction (e.g. a primate which has just eaten a fruit could potentially leave in any direction). The different turning angles (*a*_*R *_and *a*_*A*_) then represent an intensity of response to different social contexts, in this case crowding/proximity and isolation. On the other hand, in order to reduce the number of evolving parameters, we fixed *z*_*L *_and set the attraction zone to overlap with the alignment zone to allow the evolution of *z*_*A *_<*z*_*L *_and *z*_*R *_>*z*_*L*_. Aligning to neighbors without attraction is therefore not possible. We tested the implications of this simplification, by running additional evolutionary simulations in a model where *z*_*L *_could evolve and did not overlap with *z*_*A*_, and *z*_*A *_started from *z*_*L *_(see section 5 in Additional file 1). We obtain qualitatively similar results with this model indicating that our results are robust to this change in grouping rules (see Results section). The parameter range of the more traditional grouping models is a subset of the parameter range of this latter model, which implies that our model allows for a larger range of evolutionary possibilities. Moreover, we do not explicitly include noise in our model (e.g. during movement). We tested the implications of this and found that our results were robust to the addition of noise (see Results section).

### Analysis

We ran 10 evolutionary simulations for 1000 years for each of the 5 environments. We then identified evolved genotypes by conducting ancestor traces on evolutionary simulations starting with the final population and backtracing to the beginning. We extract ancestors from the end of the simulation (between year 800-900) for all simulations and plotted distributions of different parameters to identify trends and relationships. We did not take the final populations (years 900-1000), in order to exclude recent mutants which do not necessarily reflect selected genotypes. We implemented evolved parameters and visually inspected behavior of individuals by simulation and identified foraging and grouping patterns. To study why parameters evolved to certain values we used shorter "ecological" simulations (studying one genotype at a time, no mutations) and invasion simulations (studying the "invasion" of one genotype into another, no mutations). Ecological simulations allowed us to study how evolved genotypes foraged and interacted with a given environment. Invasion simulations allowed us to study whether and how a given genotype could invade a population of another genotype, and how they compare in fitness. For speed reasons we used simulations with half the field size and population. We ran 10 simulations in each case and measured food intake, inter-patch travel, intra-patch visits, patch sizes (degree depletion), proportion of a patch depleted, and group sizes.

## Results

### Evolution of two distinct types of grouping in patchy environments

**Figure 2:**
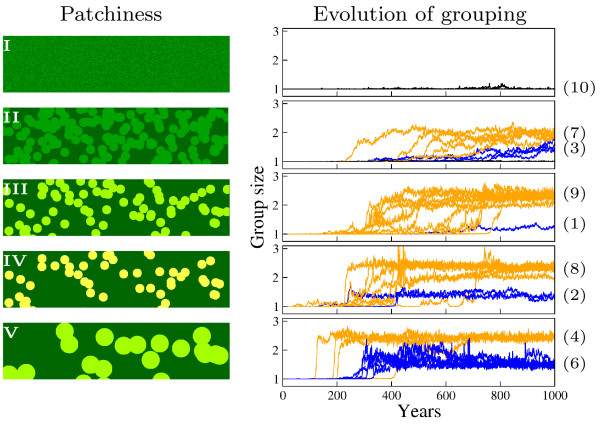
**Grouping evolves in patchy environments**. I-V: patch classes, going from uniform to increasing patchiness (see model description). Left: 1 by 0.2 km snap shots of the environments illustrating resource distributions. The more yellow the greater the density of resources; Right: Group size evolution for each of 10 simulations in each environment. Black: no grouping evolves; Orange: groups of 2-3 individuals evolve; Blue: groups size evolves to half way between solitary and small groups. Values in brackets indicate the number of simulations ending up in a particular condition.

While no grouping evolves in uniform environments (Figure 2I), we find that grouping evolves in sufficiently patchy environments (Figure 2II-V). In patchy environments we observe two possible evolutionary outcomes: (a) groups with an average size of about 2-3 individuals (Figure 2, orange lines) and (b) groups with a average size of about 1-2 individuals (Figure 2, blue lines).

**Figure 3:**
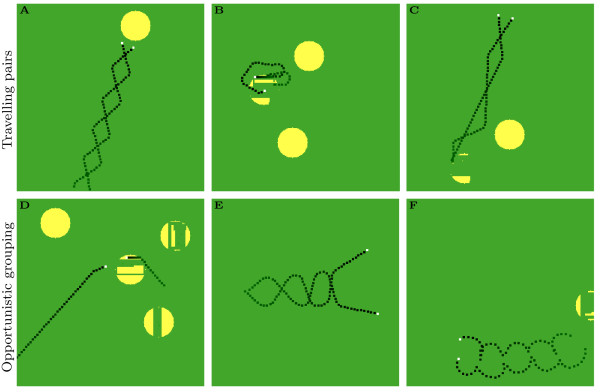
**Two types of grouping**. Top: Traveling pairs (PAIRS): (A) traveling together; (B) depleting a patch together; (C) leaving a patch. Bottom: Opportunistic grouping: (D) arriving at a patch independently; (E) splitting up "zig-zag" fashion (OPP-GRa); (F) splitting up "bouncing" fashion (OPP-GRb), after leaving a patch. White squares: individuals; Black lines: trajectory of individual fading to dark green with time; Yellow: food patches; Dark green: background (empty space). See also Animatics S1-3 in Additional file 2.

Visual inspection reveals that the two evolutionary outcomes correspond to two distinct grouping styles:

(i) **traveling pairs **(group size averages 2-3 individuals, Figure 3a-c) and (ii) **opportunistic grouping**, where individuals travel alone, but grouping rules allow individuals to aggregate towards feeding neighbors once individuals meet by chance on a patch (Figure 3d), and split up again some time after leaving the patch (Figure 3e and 3f). Opportunistic grouping tends to become more prevalent with more extreme patchiness (Figure 2V, 6 out of 10 simulations). The different types of grouping are clear in patch classes III-V, but less clear in class II, where grouping evolves later and there is less clear distinction between large and smaller groups (blue and orange).

**Figure 4:**
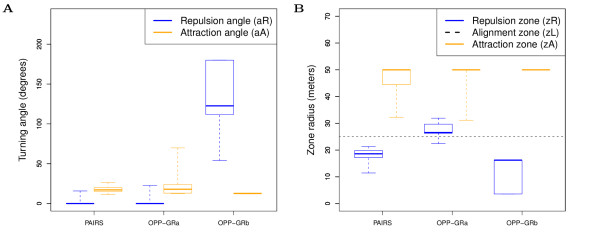
**Evolved grouping parameters in patchy environments**. (A) Turning angles (*a*_*R *_and *a*_*A*_); (B) Repulsion and attraction zones (*z*_*R *_and *z*_*A*_). For each graph we show traveling pairs (PAIRS) and both types of opportunistic grouping (OPP-GRa: *z*_*R *_>*z*_*L *_= 25 and OPP-GRb: *a*_*R *_>>*a*_*A*_). Blue: repulsion; Orange: attraction. We show data from year 800 to 900 from 10 ancestor traces in each case from patch classes III-V. Box plots show, median, upper and lower quartile, and whiskers show max and minimum values.

The different grouping styles are achieved by evolutionary adjustment of the zone of repulsion (*z*_*R*_) relative to the zone of alignment (*z*_*L*_) and the angle of repulsion (*a*_*R*_) relative to the angle of attraction (*a*_*A*_). To determine evolved parameter values, we analyze data from ancestors between year 800 and 900 from different patchy environments sorted into the different grouping style classes (as identified from average group size and visual inspection). We focus on the three most patch environments (class III-V) because of their strong evolutionary signal. Patch class II was left out because grouping emerged quite late, giving a weak evolutionary signal. In Figure 4 we show the parameter values corresponding to the following different grouping styles:

• **Traveling pairs (PAIRS)**: these individuals have a relatively low angle of attraction (Figure 4a, left orange), no angle of repulsion (Figure 4a, left blue), and a repulsion zone smaller than the alignment zone (*z*_*R *_< 25, Figure 4b, left blue). Individuals therefore partially align when close enough, but ignore each other within the repulsion zone (i.e. "tolerance zone"). Moreover the number of neighbors tolerated in the repulsion zone (*n*_*R*_) evolved to below 1 (see Section 2 in Additional file 1), which ensures that individuals are only attracted to others when alone, generating groups of 2.

• **Opportunistic grouping (OPP-GR) **is either achieved by (a) "zig-zag": having a larger repulsion zone than alignment zone (OPP-GRa, *z*_*R *_>*z*_*L*_, Figure 4b, middle blue and dashed line), or (b) "bouncing": having a larger repulsion angle than attraction angle (OPP-GRb, *a*_*R *_>>*a*_*A*_, Figure 4a, right blue and orange). In both cases, individuals are either attracted or repulsed, but do not align. The rules ensure individuals can approach another individual when it is stationary (i.e. eating), but causes splitting up when both individuals are moving. Splitting up happens because individuals do not align, leading to amplifying repulse and attraction cycles (Figure 3e and 3f).

### Why does grouping evolve?

**Figure 5:**
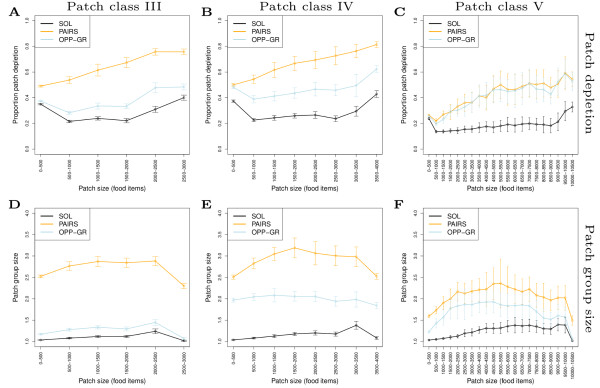
**Patch depletion for different patch sizes in different environments**. Left to right: patch class III-V. (A-C) proportion of patch depleted by groups per patch visit; (D-F) group size per patch visit. Black: solitary individuals (SOL); Orange: traveling pairs (PAIRS); Light blue: opportunistic grouping (OPP-GRa, *z*_*R *_>*z*_*L *_= 25). Each point shows mean and standard deviation of patch depletion or group size per patch visit, occurring over 10 years for 65 individuals in ecological simulations.

Grouping evolves because it allows individuals to deplete patches more efficiently. In Figure 5a-c we show patch depletion per patch visit for different sized patches as generated by partial patch depletion. In both patch class III and IV, traveling pairs are the most efficient patch depleters (Figure 5a and 5b, orange). As the environment becomes more extreme, (patch class III→V) both grouping styles (orange and blue) converge and in large patches are roughly equally good. Relative to solitary individuals, the effect of grouping is greatest with respect to intermediate sized patches.

Grouping increases overall patch depletion because it allows better "sensing" of patches. As we have shown previously, solitary foragers can detect a patch as long as individuals find food and move from food item to food item [23]. We refer to this process as TODO-based pattern recognition [29], where individuals respond and orientate themselves locally by "doing what there is to do" (scanning for and moving towards food if there is food available, otherwise moving forward). On longer timescales, this allows individuals to, at least partially, detect resource patches that are beyond an individual's local perception. This process breaks down as soon as individuals inadvertently move out of a patch, or if there are gaps in food presence, i.e. when patches are fragmented due to depletion (see Animatic S1 in Additional file 2). However, if individuals are in groups, it is possible that one of the group members is still in the patch and remains localized because it is eating. In that case, the grouping tendency of other individuals will cause them to approach the eating individual, and so automatically return to the patch and find more food (see Figure 3b and Animatic S2 and S3 in Additional file 2). Note that grouping is sufficient, individuals do not need to observe that the other individual is eating. Grouping therefore allows individuals to indirectly, or implicitly, share information about the location of food in a patch. Individuals can therefore prolong patch visit times and increase the proportion of a patch depleted.

**Figure 6:**
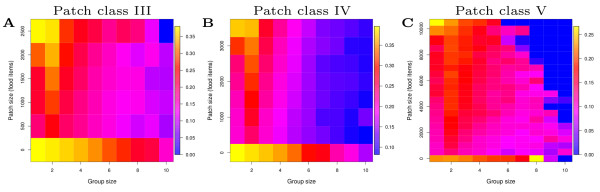
**Per capita food intake for different group sizes in different patch sizes**. (a-c) Patch classes III-V respectively. For traveling pairs (PAIRS). Proportion patch depleted by an individual measured according to how many individuals were in a patch during a particular patch visit. Each grid square is the average of all patch visits of a certain patch and group size combination over a 10 year period in simulations with 64 individuals. Note that for very large group sizes (especially in patch class V), there are fewer data points and increased sampling error but the trend is qualitatively the same as in the other environments.

Groups of 2 are sufficient for enhanced patch sensing and depletion. In Figure 5d-f we show group sizes in different patch sizes for the different environments. Per patch visit we measure how many individuals were present (Note that this excludes average group size during inter-patch travel). Thus for traveling pairs overall, and for opportunistic grouping in more patchy environments, groups sizes in patches tend towards 2-3 individuals. (Note that for opportunistic grouping, average overall group size is lower as individuals travel alone from patch to patch.) With such group sizes, patch depletion is already at 60-80% (Figure 5a-c), indicating that there is not much room for larger group sizes to contribute to enhanced food uptake. In Figure 6 we plot the per capita patch depletion (food intake) of individuals in different sized groups for patch classes III-V for traveling pairs, where blue color is the lowest value and yellow is the greatest value. For each patch visit we record how many individuals are present, how much food is in the patch (patch size), and how much food is eaten by each individual. Results reveal that group size 2 has the greatest overall per capita food intake when considering all patch size classes (second column in each figure). Solitary individuals have greater per capita intake for new unfragmented patches (top of first column in each figure), but have a lower foraging efficiency overall. On the other hand, groups larger than 2 tend to reduce per capita uptake because food has to be shared amongst more individuals, undermining the benefits of grouping. This maximal average per capita food intake (with respect to patch depletion) for groups of two, corresponds well the evolved group size (overall average group size in Figure 2, and the group size in patches in Figure 5), i.e. the observed evolutionary attractor. A group size of two therefore optimizes the trade-off between benefits of grouping and reduced food intake due to sharing patch contents with more individuals. (Note that while in this case small groups evolve, in other simulations where we add a predator, much larger groups evolve (unpublished data). Groups size is therefore not limited by the specific grouping rules in our model.)

While traveling pairs (PAIRS) are usually with 2-3 individuals within a patch in all environments (Figure 5d-f, orange), opportunistic groupers (OPP-GR) only increase in group size with more extreme patchiness (Figure 5d-f, blue). Like solitary individuals (Figure 5d-f, black), opportunistic groupers can only aggregate on patches by chance. Such aggregation mainly happens when patches become increasingly rare (Figure 5f, black). However, the patch dependent grouping of opportunistic groupers prolongs patch visits, allowing individuals to further accumulate group size in patches as compared to solitary individuals. Thus, opportunistic groupers can better deplete patches. In Section 3 in Additional file 1 we show how this enhanced aggregation can work in a more simplified mathematical model. This simplified model also shows that as patches become sufficiently rare, opportunistic grouping can be as efficient, or more so, than traveling pairs, which is why opportunistic grouping evolves mainly in patch class V (Figure 2V). Our results therefore reveal the following rule of thumb: when patches are common, travel together because, when you next find a patch, you are unlikely to find someone to deplete the patch with. However, when patches are rare, you are likely to meet your next depletion partner at the next patch, so you don't have to bother traveling together. This also emphasizes that grouping does not evolve to enhance patch finding rates, but rather to "stay in patches" in order to deplete patches better.

The simplified mathematical model also implies that slower inter-patch travel due to grouping (i.e. due to zig-zag travel, see Figure 3A), may affect how the two grouping styles relate. However, opportunistic grouping should still arise if the efficiency of solitary and group travel is equal (Section 3 in Additional file 1). Indeed in our alternative grouping simulation model, where the alignment zone can evolve and individuals in groups evolve to move in parallel (thus avoiding increased travel distance due to zig-zagging), opportunistic grouping also evolves, although less frequently (see Section 4 in Additional file 1). Moreover, average group size is the same. The finding of two types of grouping and the average group size are therefore not dependent on a zig-zag mode of travel, but are robust and generalize to an increased degree of evolutionary freedom of grouping rules.

### How does grouping evolve?

**Figure 7:**
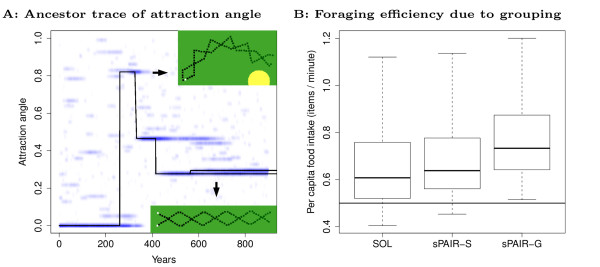
**How grouping evolves**. (A) Example of ancestor trace of attraction angle (*a*_*A*_) over evolutionary time. Blue: overall population distribution over attraction angle (*a*_*A*_); Black line: line of descent; Insets: turning groups, tPAIRS (top); straight moving groups, sPAIRS (bottom); Arrows indicate the relevant angle of attraction associated with these group movement patterns. (B) Per capita foraging efficiency of solitary (SOL), grouping individuals that only follow solitary individuals (sPAIR-S), and grouping individual that only group with each other (sPAIR-G). We measure average food items eaten per minute taken from 50 year samples of food intake from 30 SOL and sPAIR-S and 60 sPAIR-G individuals. Thus we compare 30 SOL-sPAIR-S pairs and 30 sPAIR-G-sPAIR-G pairs. sPAIRS-S represent the situation of a single grouping mutant when the first grouping mutant invades (e.g. year 250 in (A)). sPAIR-G represents the situation when multiple grouping individuals form groups. The latter case leads to an increase in foraging efficiency relative to sPAIRS-S revealing that mutual grouping generates a synergy. Box plots represent the median, upper and lower quartiles, and whiskers represent the full range of values. The solid horizontal line is the point at which energy intake due to feeding equals energy loss due to metabolism.

When a mutant genotype arises, its fitness depends on how it interacts with an existing population, both indirectly via environmental structuring (resource depletion), and directly via grouping. From ancestor traces we observe that in some evolutionary simulations when grouping first evolves, the grouping genotype actually forms pairs which frequently change direction (turning pairs, see Figure 7a, top inset). These genotypes are characterized by a large angle of attraction (*a*_*A*_). Later, smaller angles of attraction evolve, which lead to a more coordinated and symmetric "zig-zag" of individuals, causing the group as a whole to move straight (Figure 7a, bottom inset). To gain further insight into this evolutionary process we use invasion simulations to study how individuals forming turning groups (tPAIRS) and straight moving groups (sPAIRS) invade populations of solitary individuals in patch class III. Similar results are obtained in the other patchy environments.

**Figure 8:**
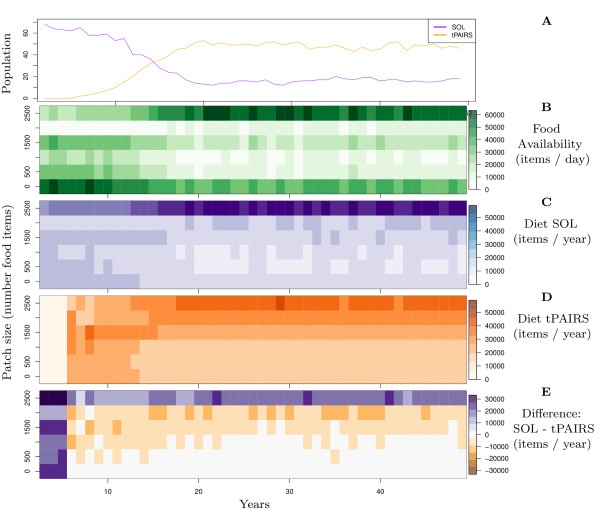
**Invasion of pairs into solitary world**. (A) Population size. Orange: traveling pairs forming turning groups (tPAIRS); Purple: solitary (SOL). (B) Distribution of food in patches of different size and fragmentation (green). (C) Food intake of solitary individuals from different patch sizes (purple). (D) Food intake of pairs from different patch sizes (orange). (E) Difference in food intake between solitary and and grouping individuals (*C *- *D*). Blue: solitary eat more; Orange: pairs eat more. Environment: Patch class III. Each grid square represents a year average.

Surprisingly, for the case of turning pairs (tPAIRS) invading solitary individuals, we find coexistence on the single resource. In Figure 8 we show details of a simulation where tPAIR individuals invade a population of solitary individuals. Initially there are only solitary individuals (Figure 8a, purple line), which obtain their food mainly from large patches (Figure 7c, top row). Large patches are therefore rare and the world is filled with partially depleted patches (see Section 5 in Additional file 1 for a mathematical description of how to see different patch sizes as a kind of "food web"). The first tPAIR individual invades after year 5, and has its peak food intake from an intermediate patch size (Figure 8d, middle rows). Initially, tPAIR individuals eat more, reproduce faster and invade (Figure 8a, orange line), because these individuals can efficiently exploit intermediate-sized and small patches. Solitary individuals are therefore out-competed on these patch sizes and their population declines. As a consequence the ecology changes: (i) the amount of food in intermediate and small patches declines (Figure 8b, bottom four rows), and (ii) the amount of food in big patches increases (Figure 8b, top rows). The latter is because tPAIRs turn a lot which makes them less successful at finding the rare large patches than solitary individuals (see [23] for how turning affects foraging success). The increasing amount of food in large patches therefore makes the world increasingly favorable for solitary individuals, so that the relative difference in foraging success between grouping and solitary individuals balances out. At this point, solitary individuals obtain more food from large patches (Figure 8e, top row), while grouping individuals obtain more food from intermediate patch sizes (Figure 8e, middle rows) and both types coexist (Figure 8a, orange and purple line: we show coexistence up to year 50, but it continues indefinitely).

This coexistence is not evolutionarily stable, because groups evolve to move straight (i.e. sPAIR individuals invade), allowing better finding and depletion of large new patches. Niche differentiation with solitary individuals is therefore reduced, preventing coexistence (see Section 6 in Additional file 1 for more detail). However, the coexistence brings into focus how niche differentiation is possible on a single resource through specialization on patches that differ in degree of depletion, or fragmentation (for further discussion see Section 5 in Additional file 1). Hence we can understand that sPAIR individuals can outcompete solitary individuals because the shift in niche allows access to a larger set of resources: those in partially depleted patches.

Since, it likely that perfectly straight moving pairs depends on the lack of noise in our model, we tested whether adding noise on the forward direction of MOVE mattered. We did this by drawing a random turning angle from a normal distribution with a given standard deviation. For the latter we consider 0.05, 0.1 and 0.2 radians and ran 10 invasion simulations for each case. In all cases sPAIRS outcompeted solitary individuals (results not shown). Noise therefore affects both solitary and grouping individuals and does not change the differences between them.

To explicitly reveal the fate of invading grouping individuals, we ran simulations where we compared solitary individuals (SOL), grouping individuals that only follow solitary individuals (sPAIR-S), and grouping individuals that only group with each other (sPAIR-G). We measured their per capita foraging efficiency when they compete together in the same environment. The results reveal that grouping individuals following solitary individuals (sPAIR-S), already have somewhat greater food intake rates than solitary individuals (Figure 7b, see also Section 7 in Additional file 1). Since solitary individuals cannot gain any advantage by being followed, the grouping individual can be termed "selfish". Moreover grouping individuals do not need to assort with other grouping individuals to have a selective advantage. However, with multiple grouping individuals, positive assortment emerges spontaneously, because grouping individuals mutually attract (see Section 7 in Additional file 1). This mutual attraction leads to enhanced foraging efficiency: two grouping individuals achieve greater foraging efficiency than grouping individuals following a solitary individuals (Figure 7b, sPAIR-G is greater than sPAIR-S). Note that while the foraging efficiency of solitary individuals (SOL) and their followers (sPAIR-S) extend to below the point at which energy intake equals energy expenditure (horizontal line), this does not happen for the mutual groupers (sPAIR-G). Thus we observe that when two "selfish" individuals get together, this can generate a mutually-beneficial system through the emergence of a synergistic process.

Fitness now depends on 'group-level" information processing about the location of food to enhance food intake. Thus during the invasion of grouping there is a transition from individual-level to multi-level information processing. Novel selection pressures arise due to the group-level structure in the population and natural selection becomes a multi-level process. Such a novel higher level selection pressure is revealed in the case of straight moving pairs (sPAIRS) invading a population of turning pairs (tPAIRS) (see Figure 7a). Here selection acts on group-level turning: it selects individuals that are more often in groups that travel straight, even though on the individual-level everyone is turning (i.e. "zig-zag") and group-level behavior is undefined. In this case, no assortment between the two types of grouping is needed: it is sufficient that sPAIR individuals spend at least some time in straight moving groups, while tPAIR individuals cannot do so (see Section 8 in Additional file 1).

## Discussion

Our results show that when individual-level sensing of a patch is difficult, grouping can evolve to generate a group-level sensing mechanism and increase foraging efficiency. While most group foraging theory focuses on the role of grouping relative to "patch finding rates" [5,6,8-11,14], our results emphasize a role of grouping relative to "staying in a patch" in order to better deplete a patch. Crucial for our results is the partial depletion of patches, because (i) it leads to a structured environment with many partially depleted patches of different size, and (ii) this allows grouping to "find its niche".

Partial patch depletion is a direct result of our focus on local information processing: the local range of detecting food items is limited relative to the size of patches. Due to these individual-level sensing constraints there is a build up of resources in partially depleted patches. Grouping releases individuals from these constraints by generating patch sensing on a larger spatial scale, which allows grouping individuals to access the resources accumulated in partially depleted patches. This shift in niche due to grouping is explicitly revealed by the co-existence between solitary individuals and turning groups. Thus partial patch depletion generates novel niche opportunities, in a process related to niche construction [30,31] or niche creation [32]. In particular, we observe how niche differentiation on a single resource can occur through specialization of the spatial scale of resource patterns that individuals can detect. These results have parallels with models that show that animals of different body size can co-exist on a single resource because of different scales of perception [33,34]. The finding of (temporary) coexistence is crucial for revealing what niche differentiation means in our model, and therefore explains how grouping can lead to enhanced foraging efficiency (i.e. also in the case of straight moving groups, a shift in niche is required for it to outcompete solitary individuals). Moreover, these results suggest an exciting potential for differences in sociality between species playing a direct role in niche differentiation. 

The evolved grouping behavior is cooperative in the sense that it leads to a mutually-beneficial outcome (i.e. a synergy), and is therefore distinct from competition. This is possible due to an implicit sharing of information that self-organizes from an interaction between foraging, grouping and the environment. This happens as follows: Eating individuals provide information about spatially aggregated resources items. Any grouping neighbors that have not found food automatically approach the feeding individual, without needing to observe that it is feeding. Other food items in the vicinity can then be found. Thus by means of grouping, the local availability of food can be implicitly (or indirectly) detected by using information generated by feeding individuals. Important is that the success of this information sharing depends on its duration. Therefore, following a solitary individual does increase a grouping individual's foraging efficiency, but this increase is limited because patch visit durations are cut short if the solitary individual leaves the patch. This happens because there is only a one-way transmission of behavior. In contrast, two grouping individuals mutually attract and information transmission becomes a two-way, or group-level, process. This generates a positive feedback, making the process self-reinforcing: one individual provides information for another individual to find food, which in turn provides information for the former to find food, and so on. This process depends on the spatial aggregation of resource items, which makes subsequent food finding events non-independent. Two grouping individuals are therefore better able to "sense" the patch and prolong the patch depletion process. In this way they gain more food per capita than they would when foraging alone, or when following a solitary individual. This synergy makes the process mutually-beneficial, which is why grouping is selected during evolution. These characteristics correspond with recent definitions of cooperation [35-37].

The mutually self-reinforced information sharing differs from the one-way information transmission generally considered in information sharing (IS) and producer-scrounger (PS) models. In IS and PS models, each patch finding event is assumed to be independent and individuals are assumed to join a patch finder [5,6,8-10,14]. In this setting, grouping can only lead to enhanced food finding rates if patches are sufficiently ephemeral, or individuals abandon patches when they are satiated [5,6,11]. Moreover, reciprocity of information production is not guaranteed. One-way information transmission is therefore inherently parasitism prone, as revealed by producer-scrounger models [8,12,13]. In contrast, our results emphasize that information sharing can occur implicitly through the grouping process itself. This could be referred to as local enhancement [38], but the process we describe is more implicit and elementary than types of local enhancement where individuals respond to seeing that neighbors are eating. Important is that the spatial aggregation of resource items leads to an automatic and immediate "reciprocity": if one individual produces information for another to eat, that eating in turn generates information for the former and so on. This process breaks down as soon as any individual stops grouping, or foraging, but this affects both individuals equally. There is therefore no incentive, nor possibility, to parasitise this process.

Through implicit information sharing, groups can detect large scale environmental patterns. While we find that small groups evolve for cooperative patch depletion, it is possible that in environments of greater complexity, such as patchy environments with greater diversity of resource types and/or quality, larger groups would be required in order for fitness relevant patterns to be detected via implicit information sharing. Moreover, such implicit information sharing can operate even when grouping evolves for other reasons, such as predation pressure, and could be very general. Indeed implicit information sharing may also be relevant for the case of well-defined and detectable "discrete" patches, such as those of IS and PS models (which in our model would be "food items that can be shared by several individuals"). This would require that patches are aggregated, but on a spatial scale that exceeds individual perception, and that at least a few patches fall within the range that individuals can detect each other (i.e. the spread of the group). If one does not directly assume an explicit social information use where all individuals will join a patch finder (e.g. joining may be restricted due to visibility constraints, or monopolization), then an implicit grouping process could lead to a situation where the finding of one patch by some individuals increases the chance of other individuals finding another patch and so on. Indeed, we already partially see this in terms of fragmented patches (a group of smaller patches), which groups are much better able to sense and deplete. However, in many group foraging models the spatial distribution of patches is undefined [5,6,8,9,14], or random and not explicitly considered [10,11]. This either prevents or limits the potential role of implicit information sharing. To find out how relevant implicit information sharing is for real group foragers requires a fine-grained approach to determine whether (i) the range at which animals can detect food is both smaller than (ii) the range at which they can sense patches or detect other patches, and (iii) the range at which they can detect neighboring individuals. Crucially, the group must be able to spread across a larger range of the resource pattern than what individuals could sense individually.

In terms of sociality and cooperative behavior, our results reveal how group foraging can generate a mutually-beneficial cooperative process. Crucially, group foraging leads to a synergistic effect. Synergy is an important prerequisite for adaptive cooperation. However, evolutionary game theory and other evolutionary approaches to cooperation do not address where such synergy comes from, but directly assume it [39-44]. We show how a mutually-beneficial synergistic process can come into existence as a side-effect of a new level of organization in the population, namely grouping. That cooperative patch depletion is a side-effect, becomes clear if one places individuals with evolved grouping rules in the uniform environment. This would not generate a mutually-beneficial process, only increased local competition for food. This emphasizes that while grouping is encoded in genes in our model, cooperation is not. The cooperative behavior is therefore not an individual-level property (strategy), but an emergent group-level property. In a patchy environment, evolution effectively only has to "invent" grouping, and then gets cooperative foraging "for free" as an evolutionary novelty. This side-effect then generates a novel selection pressure that causes grouping to be selected. In this way, understanding the origin of this cooperative behavior from the organization of individuals into groups, requires taking into account higher levels of organization and their impact on natural selection, i.e. multi-level selection. This emphasizes the role of self-organization in allowing novel phenomena, and their impact on fitness, to emerge during the evolutionary process [45-47].

## Conclusions

Our results show that the evolution of grouping can lead to enhanced foraging efficiency if groups can cooperatively "sense" patterns in the environment. In particular we show how grouping enhances depletion of food patches. Such cooperative foraging changes the niche specialization of individuals, allowing them to access resources in partially depleted patches. This reveals a role that sociality could play in ecological diversification. We find that the cooperative patch depletion emerges from self-organized information sharing, and generates novel selection pressures at the level of the group. Moreover, since individuals cannot parasitise this cooperative process, our results emphasize the origin of sociality and cooperation outside of cooperative dilemma settings, as suggested by others [48]. Instead, cooperative dilemma settings with a potential for "cheating", could be a more derived evolutionary state for social foragers, or requires extra behavioral dimensions (e.g. aggression). Our results provide a useful baseline for exploring these issues.

## Abbreviations

IS: Information sharing; MTF: Move to food; OPP-GR: Opportunistic grouping; OST: Optimal search theory; PAIRS: Traveling pairs; PS: Producer-scrounger; SOL: Solitary

## Authors' contributions

DP developed the model and conducted the analysis. DP and DS wrote the paper. All authors read and approved the final manuscript.

## Supplementary Material

Additional file 1 **Supporting information**. The first file is Additional file1.pdf in PDF format which can be viewed in any PDF-viewer such as Acrobat Reader. This file includes additional detail about the main simulation model and the modeling methodology, additional analysis, as well as details and analysis on alternative models. Click here for file

Additional file 2 **Movies in mini-website**. The second file is Additional file2.zip, which is a ZIPPED folder "miniwebsite" with file index.html (HTML) in which GIF animations can be viewed. The GIF animations reside in subfolder "miniwebsite/movies". The html file (index.html) can be opened with any web-browser and shows links to three pages showing the GIF animations: (i) Animatic S1: Solitary foraging, (ii) Animatic S2: Traveling pairs foraging, and (iii) Animatic S3: Opportunistic grouping individuals that are foraging. Descriptions of the movies are given in "index.html". These animations should start playing as soon as the link to the page with the animation is opened using a web-browser. Click here for file
