# Supplementary material for: Patch depletion, niche structuring and the evolution of co-operative foraging
Source: BMC Evol Biol. 2011 Nov 17;11:335. doi: 10.1186/1471-2148-11-335 (PMC3306211; doi:10.1186/1471-2148-11-335)
Supplement: Additional file 1 — Supporting information. The first file is Additional file1.pdf in PDF format which can be viewed in any PDF-viewer such as Acrobat Reader. This file includes additional detail about the main simulation model and the modeling methodology, additional analysis, as well as details and analysis on alternative models. [file 1471-2148-11-335-S1.PDF]

Additional file 1 for: "Patch depletion, niche structuring and the evolution of cooperative foraging"

Daniel J van der Post*[1,2,3], Dirk Semmann[1]

[1]Courant Research Centre Evolution of Social Behaviour, Georg-August Universität Göttigen, Kellnerweg 6, 37077, Göttingen, Germany
[2]Institute of Artificial Intelligence, University of Groningen, P.O. Box 407, 9700 AK, Groningen, The Netherlands
[3]Behavioural Ecology and Self-Organization, University of Groningen, P.O. Box 11103, 9700 CC, Groningen, The Netherlands

Email: Daniel J van der Post*- d.j.vanderpost@gmail.com; Dirk Semmann - Dirk.Semmann@bio.uni-goettingen.de;

*Corresponding author

Contents

1 Further model details **2**
1.1 Specification choices of scaling context 2
1.2 Foraging 3
1.3 Grouping 3
1.4 Life-history, reproduction and mutations 5
1.4.1 Mutation: 5
1.4.2 Initial conditions: 5
1.5 Detailed models in context: parameters and assumptions 5

2 Evolution of other parameters **6**

3 Patch density and foraging success of different grouping styles **8**

4 Alternative evolutionary model **12**

5 Environmental structuring and patch size niches **13**

6 Invasion of solitary individuals by straight moving groups **14**

7 Foraging success and assortment **15**

1 Further model details

1.1 Specification choices of scaling context

Spatial and temporal resolution: At the minimal level we define the minimal time and spatial scale. A minimal time step of 10 seconds t_{MIN} was chosen to keep the model running fast enough. Space is basically continuous as individuals and resources can be placed at any given location. For convenience we place resource items on intersections of a 1 by 1 meter lattice.

Local information processing: Constraints on movement and perception (local information processing) are defined in terms of the spatial and temporal scale. Maximum speed is defined in terms of the minimal time interval, so that the duration of moving 1 meter can never be below t_{MIN}. The maximum range of resource detection (r_D) is then chosen to be small, so that individuals have to move to detect food. Within this range resource detectability is simply maximal, where an item of food can be found during a 10 second FOODSCAN in an area of 10 meters squared (i.e. a search angle of about 270 degrees with a radius of 2 meters). The individuals' reach is then chosen to be below maximal resource detection range. This is quite a reasonable assumption (equal detection range and reach is probably a limit case). The importance of this assumption is that individuals can MOVETOFOOD, which allows individuals to track contiguous stretches of food (i.e. patches) thus allowing "patch detection" on longer timescales (see [1]).

Maximum ranges for awareness (z_M) and alignment (z_L) are chosen as follows: we assume that for awareness (the position of a neighbor) both sight and sound can be used, giving 50 meters as a reasonable range, e.g for primates. However, this range is limited relative to the full size of the world so that individuals in different groups, or solitary individuals, are isolated and cannot interact, i.e. there should be space for different grouping structures to arise. Moreover, for the alignment zone we assume a shorter range because alignment requires identifying the direction neighbors are moving. For identifying direction sound may also be used, but is probably more difficult than only identifying the position of a neighbor. The following relationships are therefore met:

$$d_R < r_D < z_L < z_M \tag{1}$$

Energy and life history: The minimal time interval sets a constraint on minimal feeding interval t_{MIN_EAT}, namely SEARCH + EAT ($t_{MIN} + t_E$) and for simplicity we set $t_E = t_{MIN}$. t_{MIN_EAT} then sets the maximal energy intake rate in relation to energy per food item E_r and energy metabolism E_m: $(E_r/t_{MIN\_EAT}) - E_m$. Moreover, the energy per food item E_r in relation to energy metabolism E_m set the maximum (average) feeding interval individuals can tolerate without net energy loss: $(E_r/t_{MAX\_EAT}) = E_m$ therefore $t_{MAX\_EAT} = E_r/E_m$. The energy required to reproduce $E_M/2$ defines the minimal birth interval: $E_M/2((E_r/t_{MIN\_EAT}) - E_m))$. Thus the latter four parameters define the minimal birth interval as well as minimal and maximal eat intervals.

By setting E_r, E_m and $E_M/2$ in relation to each other ($E_m < E_r <<< E_M/2$), we specify that: individuals must consume in the order of at least 10000 food items to reproduce, with a minimal birth interval of 14 days. This birth interval is quite low for mammals, but is never achieved because individuals have to move to find food and global depletion of resources lowers the foraging rate so that birth intervals increase to months-years. The reproductive cycle is therefore long enough to ensure that individuals can travel to and consume food from many patches and experience the full scale of environmental patterns in the environment. Life expectancy is roughly 10 years due to a death rate of 0.1 per year, chosen so that individuals can experience multiple reproductive cycles.

Here the relationships are:

$$t_E >= t_{MIN} \tag{2}$$

$$t_{MIN} + t_E < E_r/E_m \tag{3}$$

$$E_m < E_r <<< E_M/2 \tag{4}$$

$$E_M/2((E_r/t_{MIN\_EAT}) - E_m)) >> t_{MAX\_EAT} \tag{5}$$

Environment: The environmental settings were chosen to support a viably evolving population at low enough densities to make grouping an issue (i.e. not just individuals aggregating by chance), and to be able to specify various patchy resource distributions. Population size (carrying capacity) is defined in terms of the maximum feeding interval in relation to resource influx into the environment: $t_{MAX\_EAT} * R_g$ (i.e. R_g divided by the minimal rate of feeding). Thus population size can be directly linked to the ratio E_r/E_m. Resource influx rate gives the total amount of resources per year R_T. The size of the world A then gives the max density of resources R_d. Within the constraints of A and R_T we distribute resources in different ways. Besides $A >> 0$, the following relationship is met to obtain the desired population size:

$$t_{MAX\_EAT} * R_g > 100 \tag{6}$$

1.2 Foraging

We use the evolvable foraging algorithm we previously referred to as "extended" in van der Post & Semmann [1]. Individuals have (i) a decision making algorithm and (ii) the details of behavioral actions (Fig. 1a and b in main text). Individuals can execute the actions MOVE, FOODSCAN, MOVETOFOOD and EAT (ovals). MOVE and FOODSCAN can alternate depending on probabilities to repeat those actions (p_M, p_{SE}, p_{SN}). MOVE and FOODSCAN cannot occur at the same time (intermittent foraging). FOODSCAN can detect FOOD or NOFOOD (rectangles). If FOOD is found individuals EAT if food is within reach ($d_R = 0.9$), or first MOVETOFOOD before EAT, moving up to half of reach from the food. After EAT or NOFOOD, individuals can choose whether to repeat search or not. Previous evolution of solitary foragers showed that individuals evolve decision rules in order to repeat FOODSCAN after finding food ($p_{SE} = 1$), while never repeating FOODSCAN after NOFOOD ($p_{SN} = 0$).

MOVE and FOODSCAN have durations, distances and angles that can evolve. In MOVE, individuals move forward with distance d_M and turn with angle a_M from their current direction, taking t_M seconds per meter. In FOODSCAN, individuals search an area defined by radius d_F, and angle a_F, for t_F seconds. Food items up to 2 meters can be detected, and detection varies with area scanned and duration. EAT takes t_E seconds. Solitary individuals evolved to search areas that maximized detection ($d_F = 2.1$, $a_F = 260 - 280$ degrees, $t_F = 10$ seconds). Move parameters varied according to the patchiness of the environment.

For each environment we use evolved parameter conditions of solitary individuals [1] as initial conditions (see Table S1).

1.3 Grouping

For grouping, we allow parameters to evolve which affect how individuals move in relation to neighboring individuals, as is done in various grouping and flocking models [2,3]. As a simplification, grouping can only

Table S1: **Initial conditions for simulations in different environments.**

**Parameter**	**Patch class**		
	I	II	III-V
p_M	0.0	0.128	0.209
t_M	10	10	10
d_M	2.0	4.85	4.5
a_M	0.0	0.01	0.0
p_{SE}	1.0	1.0	1.0
p_{SN}	0.0	0.0	0.0
t_F	10	10	10
d_F	2.1	1.91	2.01
a_F	240	310	254
p_{MTF}	1.0	1.0	1.0

happen during MOVE, i.e. movement when no food is detected. Thus, grouping tendencies do not operate continuously, but arise intermittently and context-dependent.

An individual's neighbors affect its direction of movement according to how far those neighbors are. Each individual has a position vector ($\vec{p_i}$) and a heading ($\vec{v_i}$). Individuals turn away from the position of neighbors at close distances (repulsion zone, z_R), with a maximum angle (repulsion angle, a_R), if there are more than n_R neighbors in this zone. The latter parameter is not often included in grouping models, but allows that individuals tolerate a certain number of individuals close by without reacting to them. If there are less than n_R neighbors in z_R, then individuals turn towards individuals within an attraction zone (z_A), and align with individuals within an alignment zone (z_L), with a maximal angle (attraction angle, a_A).

The preferred direction of an individual ($\vec{d_i}$) in case of repulsion is calculated as:

$$\vec{d_i} = -\frac{\sum_{j \neq i}^{n_{RZ}} \vec{r}_{ij}}{|\sum_{j \neq i}^{n_{RZ}} \vec{r}_{ij}|} \tag{7}$$

where $\vec{r}_{ij} = (\vec{p_j} - \vec{p_i})/|(\vec{p_j} - \vec{p_i})|$ is the unit vector in the direction of neighbor j, and n_{RZ} is the number of neighbors in the repulsion zone.

In the case of attraction:

$$\vec{d_i} = \frac{\sum_{j \neq i}^{n_A} \vec{r}_{ij} + \sum_{j \neq i}^{n_L} \vec{v_j}}{|(\sum_{j \neq i}^{n_A} \vec{r}_{ij} + \sum_{j \neq i}^{n_L} \vec{v_j})|} \tag{8}$$

where n_L is the number of individuals between the repulsion and alignment zone, n_A is the number of neighbors in between the repulsion zone and attraction zone and $\vec{v_j}$ is the unit direction vector of neighbor j. Once the preferred direction is calculated, individuals turn towards it with the appropriate angle. If the angle between $\vec{v_i}$ and $\vec{d_i}$ is less than the maximum turning angle, then $\vec{v_i} = \vec{d_i}$, else the individual turns (up to the maximum angle) in the direction of $\vec{d_i}$. Note that in our grouping model, the zone of attraction and alignment overlap, so that individuals cannot exclusively align to neighbors without also being attracted to them. This overlap was implemented as a model simplification in order to fix the alignment zone to reduce the number of evolving parameters, without the extra assumption that the repulsion or attraction zone needed to be larger or smaller than the alignment zone (i.e. thus we allow the system to evolve not to have alignment). To test the implications of this choice we also ran simulations with a second grouping model

where the alignment zone could evolve and the attraction zone was always greater than the alignment zone (see Section 4).

1.4 Life-history, reproduction and mutations

Individuals gain energy through food ($E_r = 2$ energy units per item) which is added to their energy store e_i (with a maximum: $E_M = 100000$). To survive, individuals must have energy ($e_i > 0$) and energy intake must compensate basal metabolism ($E_m = 1$, which is subtracted from e_i every minute). Individuals must move to eat, however we do not add explicit movement costs, but time spent moving cannot be spent eating.

Individuals reproduce when $e_i = E_M$. Energy is then halved and the other half goes to a single offspring. The time taken to get back to E_M defines a birth interval. Shorter birth intervals means greater rate of reproduction. Individuals can die with a probability of 0.1 per year, which adds some stochasticity in survival. We limit maximum age to 10 years.

1.4.1 Mutation:

When individuals reproduce, the parameters of decision making and behavioral actions are inherited by offspring, with a probability of mutation: $p_m = 0.05$ per parameter ("gene"). We allow all action durations, distances and angles to evolve except d_R and t_E. The mutation "step" is defined by drawing the parameter value from a normal distribution with the mother's parameter value as mean and standard deviation scaled to about 20% of the range of values that is relevant for that parameter. In order to keep simulations running fast enough, we limit the minimal action duration to $T_{min} = 10$ seconds. Most mutations are close the mother's parameter value, but larger jumps are possible. This type of mutation was chosen to make evolution of parameters possible without predefining their ranges.

1.4.2 Initial conditions:

We take evolved parameters of solitary individuals form van der Post and Semmann [1] as initial conditions (see Table S1), and therefore always initialized a viable population. These parameters were found to have evolved to enhance individuals abilities to detect patches to remain within them and deplete them, as well as in patchy environments, long move distances evolved in order to bias foraging to large patches. Here we also study new environmental conditions (increased patchiness). In those cases we initialize populations with parameters of solitary individuals evolved in the next most patchy setting (patch class III). In those simulations we observe further optimization of solitary foraging next to the evolution of grouping (see Section 2).

1.5 Detailed models in context: parameters and assumptions

Given the complexity of biological systems, all models of those systems require significant simplifying assumptions. These assumptions define how a model relates to a given biological system and how to interpret the model. Some assumptions are explicit (parameters, variables and relationships between them), and others are implicit within simplifications on spatio-temporal resolution.

For models with few parameters there is often a distinction between explicit parameters and implicit assumptions following from the model formalism (e.g. no space in ODE models, coarse-time scales). In

studying such models the focus is on the parameter space. Thus the model behavior (or outcome) can be well established. However, interpretation of the model relative to a biological system stills needs to be done in light of the implications of implicit assumptions. These implications are rarely known, because implicit assumptions are hardly ever addressed directly.

For models with a high resolution space and time, such as our model, a much larger set of explicit assumptions in the form of parameters is required to specify the model. The implications of these specific choices cannot all be addressed within a given study. Therefore there is a set of invariant parameters (cf invariant implicit assumptions of less specified models). Thus the model's behavior across the complete parameter space cannot be revealed. Relative to models with few parameters, this incomplete model analysis suggests that models with many parameters can hardly ever be thoroughly analyzed. However, this assesment is only derived if one focusses exclusively on parameters, rather than on the complete range of assumptions (i.e. both explicit and implicit), and how they relate to the biological system being modeled. In fact, the interpretation of parameters and assumptions in indivdiual-based specified models is generally more straight forward (spatial and temporal scales may be more similar, parameters can easily be interpreted in light of individual properties) relative to the biological system in question [4]. Thus, if we accept that fixed parameters of highly specified models are just part of the invariant set of assumptions (both implicit and explicit) that any model study requires, then we can take the following view:

1. All models require simplifying assumptions (both explicit and implicit) relative to a given biological system
2. Any model study cannot address all assumptions, i.e. there is always a set of invariant assumptions (unaddressed).
3. Invariant assumptions set the context in "model space" (defined by all possible model specifications) in which analysis takes place.
4. In coarsely specified models (few explicit parameters) most invariant assumptions are implicit, while in finely specified models (many parameters) invariant assumptions occur increasingly in the form of explicit parameters.
5. Only comparison across different models (and model types) can reveal the implications of invariant assumptions (e.g. see [5] for a nice example).

2 Evolution of other parameters

In Fig. S1 we show the average evolved parameters for the different grouping styles other than the grouping zones and angles shown in Fig. 4 (main text). Relative to grouping we observe that the number of neighbors tolerated in the repulsion zone (before attraction switches to repulsion) increases somewhat with opportunistic grouping (Fig. S1a, blue). This is because opportunistic grouping only occurs around and within patches, and n_R does not play a role during between patch travel. Its specific value therefore becomes less important. Note that for PAIRS, $0 < n_R < 1$, to ensure groups do not get too big. Attraction only happens when an individual has no neighbors in its repulsion zone, so that attraction stops with one partner generating groups of 2 (see also Table S3).

For foraging parameters (see Table S2 for values) we basically reconfirm trends we found without grouping [1]. We find that move distance increases somewhat with opportunistic grouping because this

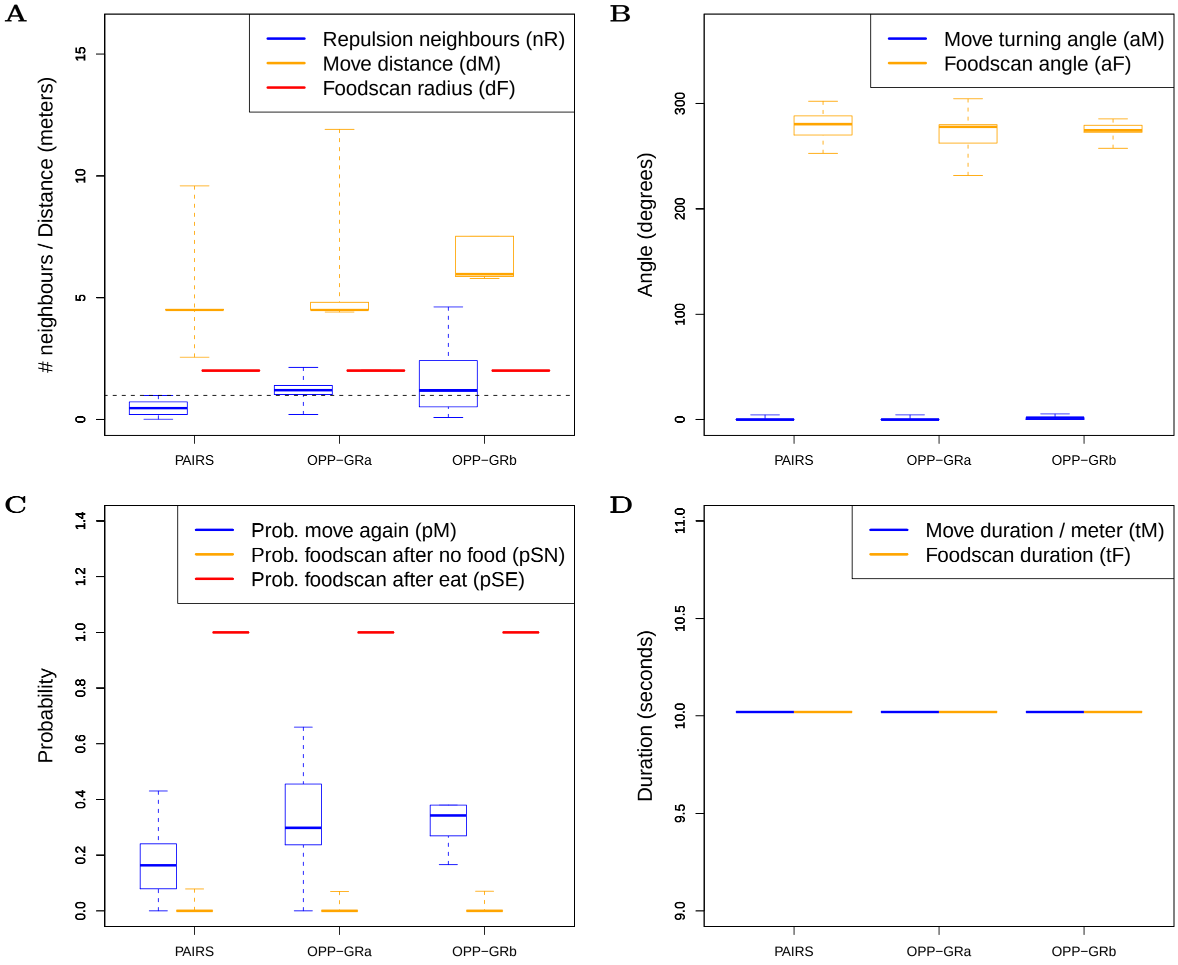

Figure S1: **Evolved parameters in patchy environments**. (a) Repulsion neighbors and foraging distances (n_R, d_M, and d_F); (b) foraging angles (a_M and a_F); (c) action probabilities (p_M, p_{SN} and p_{SE}); (d) foraging durations (t_M and t_F). For each graph we show travelling pairs (PAIRS) and both types of opportunitic grouping (OPP-GRa ("zig-zag"): $z_R > z_L = 25$ and OPP-GRb ("bouncing"): $a_R >> a_A$). We show data from year 800 to 900 from 10 ancestor traces in each case. Box plots show, median, upper and lower quartile, and whiskers show max and minimum values.

coincides with increased patchiness (Fig. S1a, orange). This increase corresponds with slight increases in probability to repeat moving (Fig. S1c, blue). Previously we found that these parameter changes increase the rate at which individuals travel between patches, as well as increase the size of patches individuals stop to feed in [1].

Other parameter values are basically conserved on values previously evolved in solitary individuals. This conservation is true for FOODSCAN radius (Fig. S1a, red), FOODSCAN and MOVE angles (Fig. S1b), probabilities to FOODSCAN after not finding food and after eating (Fig. S1c, orange and red), and action durations, which stay on minimal values (Fig. S1d).

These results show that there is in fact little feedback of grouping parameters on the evolution of foraging

parameters, suggesting that foraging and grouping parameters evolve relatively independently.

Table S2: **Evolved foraging parameters.** The averages and standard deviations (in brackets) of ancestors between year 800 and 900 of all 10 simulations of all settings (which is approximately 70-80 ancestors per simulation). Angles are shown in degrees, distances in meters and durations in seconds.

**Parameter**	**Patch class**				
	I	II	III	IV	V
p_M	0.000 (0.000)	0.053 (0.066)	0.115 (0.084)	0.190 (0.099)	0.338 (0.157)
t_M	10 (0.00)	10 (0.0)	10.02 (0.0)	10 (0.0)	10 (0.0)
d_M	2.034 (0.085)	4.712 (0.538)	4.689 (0.562)	4.521 (0.158)	5.960 (2.316)
a_M	0.002 (0.012)	2.205 (2.021)	0.658 (1.237)	0.295 (1.081)	0.394 (1.026)
p_{SE}	1.570 (0.368)	1.317 (0.235)	1.347 (0.316)	1.340 (0.242)	1.400 (0.247)
p_{SN}	0.000 (0.002)	0.000 (0.004)	0.004 (0.014)	0.000 (0.002)	0.001 (0.006)
t_F	10 (0.0)	10.021 (0.008)	10.02 (0.0)	10.000 (0.000)	10.0 (0.000)
d_F	2.090 (0.026)	1.912 (0.005)	2.001 (0.000)	2.011 (0.006)	2.011 (0.005)
a_F	258.84 (6.239)	308.47 (2.516)	279.40 (10.293)	282.979 (16.567)	270.049 (8.168)
p_{MTF}	1.315 (0.286)	1.367 (0.324)	1.331 (0.253)	1.359 (0.367)	1.651 (0.402)

Table S3: **Evolved grouping parameters.** The averages and standard deviations (in brackets) of ancestors between year 800 and 900 of all 10 simulations of all settings (which is approximately 70-80 ancestors per simulation). Those parameters that differ are shown in bold. Angles are shown in degrees, distances in meters and durations in seconds.

**Group type**	**Patch Class**	**Parameter** z_R	z_A	a_R	a_A	n_R
PAIRS	III	17.416 (2.039)	54.189 (17.189)	0.015 (0.137)	17.230 (2.745)	0.531 (0.316)
	IV	18.367 (1.928)	47.947 (10.172)	2.443 (5.515)	17.232 (4.161)	0.338 (0.284)
	V	21.923 (3.051)	78.834 (15.311)	0.215 (0.693)	16.355 (2.629)	0.755 (0.380)
OPP-GRa	III	26.391 (0.085)	32.066 (0.303)	0.000 (0.000)	67.957 (2.561)	0.534 (0.576)
($z_R > z_L$)	IV	29.140 (0.816)	57.662 (18.158)	0.000 (0.000)	24.759 (14.804)	0.962 (0.440)
	V	27.630 (2.964)	70.955 (17.694)	0.184 (0.590)	17.563 (4.175)	1.403 (0.271)
OPP-GRb ($a_R > a_A$)	V	10.129 (6.343)	60.509 (5.388)	131.054 (40.626)	12.457 (0.280)	1.371 (1.074)

3 Patch density and foraging success of different grouping styles

Here we derive a simpified mathematical "toy" model to conceptualize the relationships between group food intake, patchiness in the environment, travel group size and average number of individuals in patches. First of all we express foraging efficiency as the relative time spent in patches, which is roughly equivalent to the probability than an individual is in a patch at any moment:

$$T_e = \frac{T_{wp}}{T_{wp} + T_{bp}} \tag{9}$$

If time spent in patches T_{wp} represents feeding time and depletion of patches, then the fraction of time spent eating T_e is optimized when $\frac{T_{wp}}{T_{wp}+T_{bp}}$ is maximal.

From simulations (see Results in main text), we know that patch depletion is a function of group size: time spent in a patch increases with better sensing of the patch, but is reduced through competition (sharing patch contents):

$$T_{wp} = \frac{t_{mse} P_d(N_P) P_f}{N_P} \tag{10}$$

$$P_d(N_P) = \frac{(N_P)^n}{(N_P)^n + (h_{pd})^n} \tag{11}$$

where t_{mse} is the time taken for a move search eat sequence, P_f is the total number of food items in the patch, N_P is the number of individuals in the patch, $P_d(N_P)$ is the fraction of patch depleted as function of N_P, h_{pd} is the half max patch depletion, and n is a scalar of the depletion function. Here we simply approximate the group dependent patch depletion with a sigmoidal function (Fig. S2c).
Thus for a given inter-patch travel time (T_{bp}), group size can first increase T_e because it leads to longer patch visits and more feeding, but as soon as the increase in P_d does not weigh up against N_P then grouping starts to reduce T_e again.
Next we approximate between patch travel (T_{bp}) as:

$$T_{bp} = \frac{A_T}{A_p P_N P_{fa}} \frac{t_{ms}}{d_m} D_p \tag{12}$$

where A_T is the total area of the environment, A_p the area of a single patch, P_N the total number of patches, P_{fa} is the fraction of patches available at that time, t_{ms} is the time needed for one move and search cycle, and D_p is the diameter of a patch, d_m is the distance moved per move event. We therefore simply approximate the time needed to determine whether a certain area contains a patch as scaled with moving on a diagonal across it. For simplicity we assume a fixed number of patches of the same size (i.e. we ignore effects of depletion).
A single move-search cycle t_{ms} is then defined as:

$$t_{ms} = (t_m d_m + t_s) G_{tf} \tag{13}$$

where t_m is time per meter moved, G_{tf} is a group travel factor which scales movement efficiency relative to solitary straight line movement (e.g. due to zig zagging increasing distance moved), and t_s is the time per search event. Given these expressions we can plot T_{bp} against total number of patches in the environment (Fig. S2a).
The number of individuals in a patch (needed for determine patch depletion) then depends on inter-patch travel through its effect on T_e, and is approximated as:

$$N_P = G_s + \frac{T_e(N_T - G_s)}{P_N P_{fa}} \tag{14}$$

where G_s is the size of traveling groups, N_T is the total population size, P_N is the total number of patches (as in the input parameters for our simulations), and P_{fa} is the fraction of patches available at any given time because of resource depletion. This equation approximates that as there are fewer patches, individuals are more likely to end up in the same patch as another individual. This likelihood depends on T_e (the fraction of time an individual spends in a patch, or the probability that an individual is in a patch, see above). Therefore there is an interaction between group size in patches and patch depletion, because patch depletion affects time in a patch and hence the likelihood of individuals to find each other in a patch and increase time spent there.

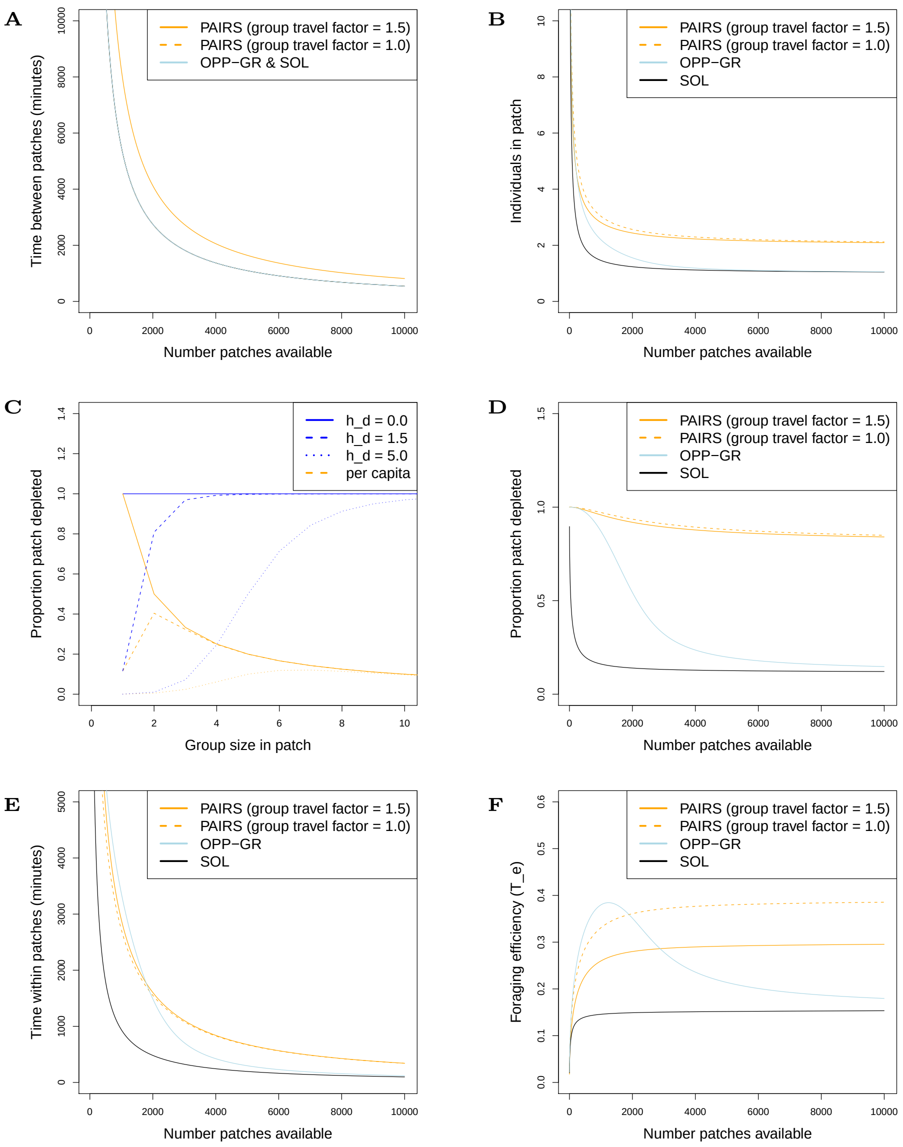

Figure S2: **Overview of results mathematical model.** (A) Between patch travel time. (B) Number of individuals ending up in patches together. (C) Group size dependent patch depletion. (D) Proportion patch depleted. (E) Within patch time. (F) Foraging efficiency. Orange: Traveling PAIRS (we include groups that travel slower (solid line) and equally fast (dashed line) as solitaries); Blue: opportunistic grouping (OPP-GR); Black: solitary individuals (SOL); Parameters as in main simulations and resource density at carrying capacity is set at 0.02, which is roughly equivalent to density in most simulations. "Number patches" (P_A) is equivalent to the input parameter of patch class (i.e. Class II = 19653, Class III = 8000, Class IV = 5333, Class V = 2000). Patch size (P_F) is then R_T/P_A, and the number of patches available at any one time is roughly $0.02 * A/P_F$. Patches available is the value we use to compute likelihood to find patches etc (see equations).

Given these simplified relationships, we can then (not particularly informatively) express foraging efficiency as:

$$T_e = \frac{t_{mse} P_d(N_P) P_f}{t_{mse} P_d(N_P) P_f + N_P \frac{A_T}{A_p P_N P_{fa}} t_{ms} D_p} \tag{15}$$

where P_f is the number of food items per patch (R_T/P_N).

We simulated this simplified model using parameters based on our simulations (Parameters are as follows: $t_{mse} = 30secs$; $t_{ms} = 20secs$; $A_T = 5660^2 m^2$; $R_T = A * 0.53$; $P_f = R_T/P_N$; $P_{fa} = 0.02 R_T/P_f$; $n = 5$; $h_{pd} = 1.5$; $D_p = 40$; $A_p = \pi \frac{D_p}{2}^2$; $d_m = 4.5$; $G_f t = 1.5$ for pairs, else 1.0; $G_s = 2$ for pairs, else 1.0; $N_T = 120$.) and approximating patch depletion so that solitary individuals deplete about 20% while pairs deplete about 80% of patches. We then varied P_N and compared solitary, traveling pairs (idealized to travel always in pairs) and opportunistic groupers. Given the feedback between the equations ($T_e \rightarrow N_p \rightarrow P_d(N_P) \rightarrow T_{wp} \rightarrow T_e$) we iterated the model until T_e reached equilibrium. We ran the model for different patch number input values (as for simulations), where the amount of food available at any one time was equal to 2% of total amount of food (roughly equivalent to the average density of resources in the simulations). The patch size was then the total amount of food divided by the number of patches given as input. The total number of patches available at any one time was the 2% of total food divided by patch size.

Results are shown in Fig. S2. In the model inter-patch travel time depends on patch density. As the number of patches decreases the inter-patch travel time increases (Fig. S2a). If we add a group travel factor G_{tf} for groups, then groups travel slower and have larger inter-patch travel times. Patch density affects the number of individuals that are likely to find the same patch by chance, even in solitary individuals (Fig. S2b, black line). However the interaction with patch depletion and patch visit times allows opportunistic groupers to increase group size faster (Fig. S2b, blue line): grouping with others on patches allows greater patch visit times allowing more individuals to group on a patch. Traveling pairs always have at least 2 individuals together on a patch.

Given the chosen input function of patch depletion according to group size (Fig. S2c, dashed line), we see that as patch density decreases the proportion of patches that are depleted increases in all cases (Fig. S2d), due to increasing group size (Fig. S2b). Thus pairs generally deplete patches well at all densities, but opportunistic groupers start to do particularly well when convergence of individuals on patches happens more often due to chance. Therefore opportunistic groupers start to spend more time in patches (Fig. S2e), and hence more time feeding (i.e. greater foraging efficiency, see Fig. S2f). Therefore, traveling pairs do best at higher densities of patches because traveling pairs always have a depletion partner, while opportunistic groupers start to do better at lower densities when depletion partners are likely to be found by chance. In this particular simplified model opportunistic grouping tends to become the most optimal at some point (Fig. S2f, blue), even relative to pairs that do not have slower travel (Fig. S2f, orange dashed). This greater foraging efficiency of opportunistic grouping is because for pairs, groups start to become too big at low patch densities (too much of the patch has to be shared). However, this specific relationship between the two grouping styles probably does not hold directly in the main simulation model because of the various simplifications made here. Our main point here is to show how a simplified extraction of the main model can qualitatively reveal the inter-relation of patch density and the success of different grouping styles. In that sense, the results of this toy model are in line with the findings from the main simulation models.

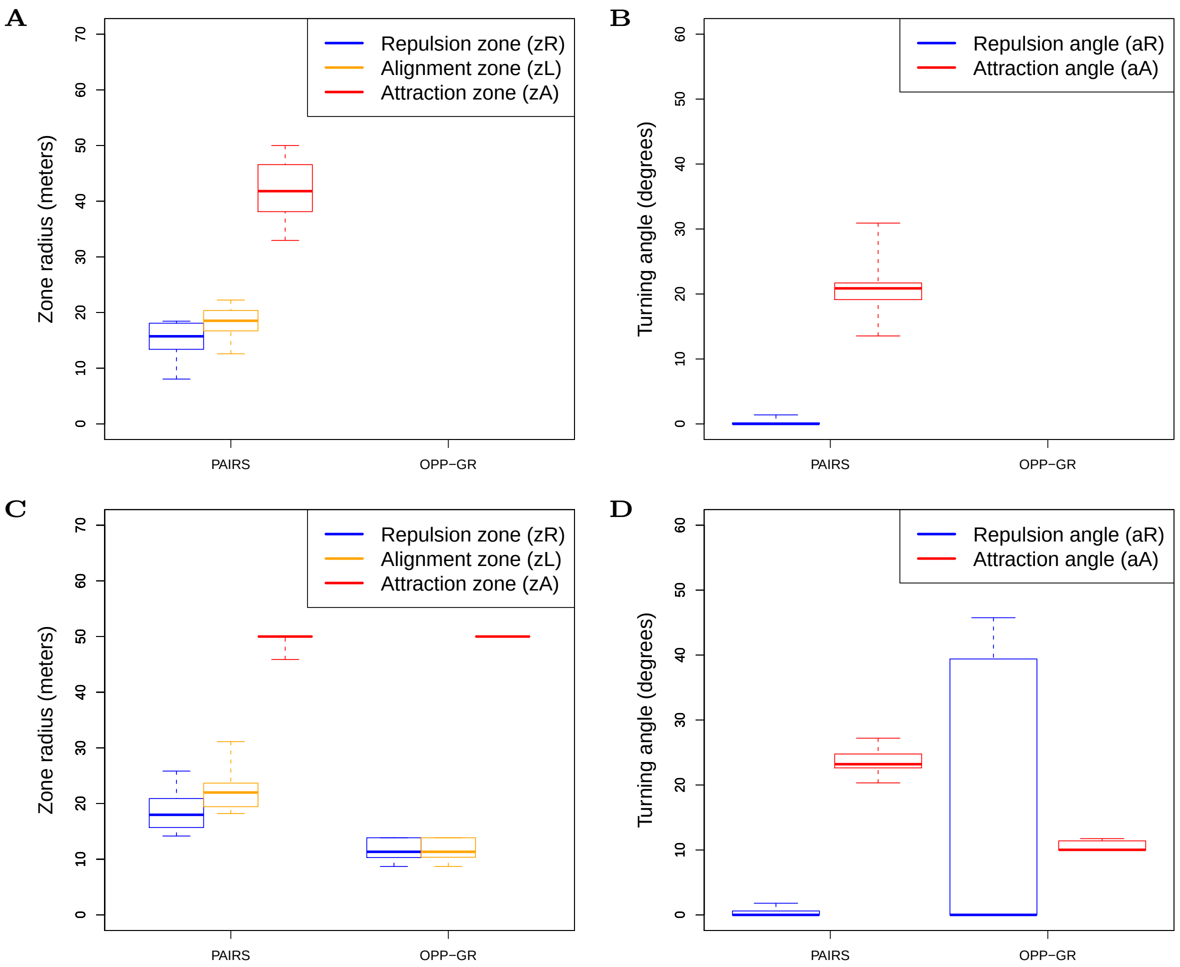

Figure S3: **Evolved grouping parameters in alternative grouping model**. (a,c) Repulsion, alignment and attraction zones (z_R, z_L, and z_A), (b,d) angles (a_R and a_A). (a-b) Patch class III, (c-d) Patch class V. For each graph we show travelling pairs (PAIRS) and opportunitic grouping (OPP-GR: $z_R >= z_L$). Blue: repulsion; Orange: alignment; Red: attraction. We show data from year 800 to 900 from 10 ancestor traces in each case. Box plots show, median, upper and lower quartile, and whiskers show max and minimum values.

4 Alternative evolutionary model

To test the implications of some of our simplifying assumptions, namely not allowing the alignment zone z_L to evolve, we tested a second model where the alignment zone z_L could evolve. In contrast to the main model, here alignment and attraction were set to be non-overlapping so that $z_R < z_L < z_A$. Otherwise grouping rules were identical to the main model (see Section 1.3 and Model description in main text).
We tested this model in environments with patch class III and V, and focused mainly on what kinds of grouping emerged, particularly whether opportunistic grouping would still arise in the more patchy environment.
Results showed that all 10 simulations in patch class III lead to traveling pair grouping. The main

difference with the main simulations was that pairs could travel in parallel (i.e. did not zig-zag). Here the pattern is to have a large attraction zones (Fig. S3a, red), and smaller repulsion and alignment zones, where the alignment zone is somewhat larger than the repulsion zone (i.e. in all cases $z_R < z_L$) (Fig. S3a, blue and orange). For angles, the repulsion angle does not evolve (Fig. S3b, blue), while the attraction angle is fairly low (Fig. S3a, red). The same pattern of parameter values is observed for traveling pairs, in the environment with patch class V (Fig. S3c-d). Both the values of evolved zones and angles are not much different from those in the main model (Fig. 4 main text). In fact evolved values of z_L are quite close to our assumed value (plus minus 20 versus 25). The parallel group travel implies a more efficient inter-patch travel rate, and reveals that the zig-zag pattern of movement was due to the restriction of the attraction and alignment zones overlapping (never only alignment). The main impact of forcing the overlap of alignment and attraction, is that groups can only travel in zig-zags. Nonetheless, although here grouping individuals travel parallel evolved group size and parameter values end up pretty much the same.

In patch class V we find that 8 simulations evolve traveling pairs, while 2 evolve to opportunistic grouping (mainly solitary travel but collective patch depletion). The latter is achieved by a large attraction zone, a small repulsion zone and no alignment zone ($z_R = z_L$) (Fig. S3c, right 3 box plots). In addition an angle of repulsion does sometimes evolve ($a_R > 0$), but there is much variation. The angle of attraction is somewhat smaller than for traveling pairs (Fig. S3d). The outcome is that there is only weak attraction, some repulsion and no alignment. This causes individuals to split up once both are moving, making grouping specific to patch contexts.

These results reveal that the finding of the two grouping styles is fairly robust to changes in degrees of freedom for the evolving grouping rules. Although there are fewer cases of opportunistic grouping (2 out of 10, rather than 6 out of 4 in the main model), the phenomena does evolve. A likely reason that it is rarer in this alternative model is that inter-patch travel is less affected by grouping (no zig-zag, but parallel movement), making the difference of pair movement with solitary movement less pronounced. Thus the relative advantage of traveling alone may only arise at lower patch densities (see also Section 3).

5 Environmental structuring and patch size niches

In our model patches are partially depleted, generating smaller patches, which in turn are partially depleted. Thus over time the foragers feed on a range of patch sizes. Thus if we view different patch sizes (or classes thereof) as different resources, we can model a kind of simple "ecosystem" or "patch-web" with ODEs (ignoring for the moment inter-patch movement dynamics):

$$\frac{dR_M}{dt} = I - eF_f R_i \tag{16}$$

$$\frac{dR_i}{dt} = \sum_{f=1}^{T} eF_f \left(\sum_{j>i>0}^{M} p_{jif} R_j - R_i \right) \tag{17}$$

$$\frac{dF_f}{dt} = c\left(\sum_{i=1}^{M} p_{jif} eF_f R_j (R_j - R_i)\right) - dF_f \tag{18}$$

where $\frac{dR_i}{dt}$ is the change in R_i, the density of patches of size i, F_f is the density of foragers of type f, T the total number of forager types, e is an encounter parameter relating inter-patch movement to encounters with patches, M the maximum patch size (category), I is the fixed influx of maximum patch size into the

system, p_{jif} the probability of converting a patch of size j to i by forager f, $R_j - R_i$ is the amount of food obtained from converting patch size j to i, c is a conversion factor defining rate of reproduction relative to food intake, and d is a fixed death rate for foragers.

From this model it is clear the matrix p_{jif} will determine both the size of the population of foragers and the frequency distribution of patch sizes. p_{jif} in fact represents how well individuals deplete patches across the whole range of patch sizes: i.e. their "spatial" niche specialization (ignoring for the moment inter-patch dynamics which further affects which individuals meet which patches). Moreover, from this description we can more easily understand how differentiation on this patch fragmentation niche allows co-existence: if the left-overs from a species specialized on large patch type are sufficient to support a population of another species better specialized on smaller patches, then co-existence can occur (see Results, main paper). At the one extreme all p_{jif} are zero, and all largest patches are fully depleted. There are no left-overs and no co-existence. At another extreme one species (A) feeds on the largest patches, depleting them by half. It is however unable to deplete those half-size patches efficiently obtaining only one item at a time. For a second species (B) the reverse is true. B rapidly consumes what A would otherwise slowly deplete over time. Thus species A "out-competes" species B on large patches, but species B "out-competes" species A on half-patches. B relies on depletion by A, but A cannot avoid generating a world pleasant for species B. At equilibrium both species can co-exist.

Of course the different resources modeled here are not really different resources, but simply the same resources with a different local spatial structure. It is the specific interaction of local information processing with different local resource configurations that allows us to conceptualize different patch size categories as "different" resources, because to foragers with different properties these resource are effectively different. Our main simulation model shows how patches of different size can effectively be different resources and lead to niche differentiation (see Results main paper).

6 Invasion of solitary individuals by straight moving groups

Here we show details of a case-study simulation of straight traveling pairs (sPAIRs) invading a population of solitary individuals (Fig. S4). Initially there are only solitary individuals (Fig. S4a, purple line), which obtain their food mainly from large patches (Fig. S4c, top row) which solitary individuals are best at depleting (see Fig. 6a, top left block, main text). Large patches are therefore rare in the environment (Fig. S4b, top row). Solitaries obtain much less food from smaller patches (Fig. S4c, bottom four rows) even though more food is available in these patch size categories (Fig. S4b, bottom four rows), because these patches are difficult to deplete (see Fig. 6a, below top left block, main text).

The first sPAIR individual is introduced after year 5, and has its peak food intake from an intermediate patch size (Fig. S4d, mid row). The difference between solitary and sPAIR feeding reveals that solitaries obtain more food from large patches (Fig. S4e, top row), while sPAIRs obtain more food from intermediate patch sizes (Fig. S4e, middle rows). This difference represents niche differentiation between the two types of foraging. However, overall sPAIR individuals are more efficient foragers (Fig. S5a, orange lines), reproduce faster and invade (Fig. S4a, orange line). As grouping individuals invade, the ecology changes: (i) the amount of food in intermediate and small patches declines (Fig. S4b, bottom four rows), and (ii) the amount of food in big patches increases (Fig. S4b, top row) because the number of solitary individuals declines. Despite the niche differentiation, solitaries are out-competed, because niche overlap on large patches is too large.

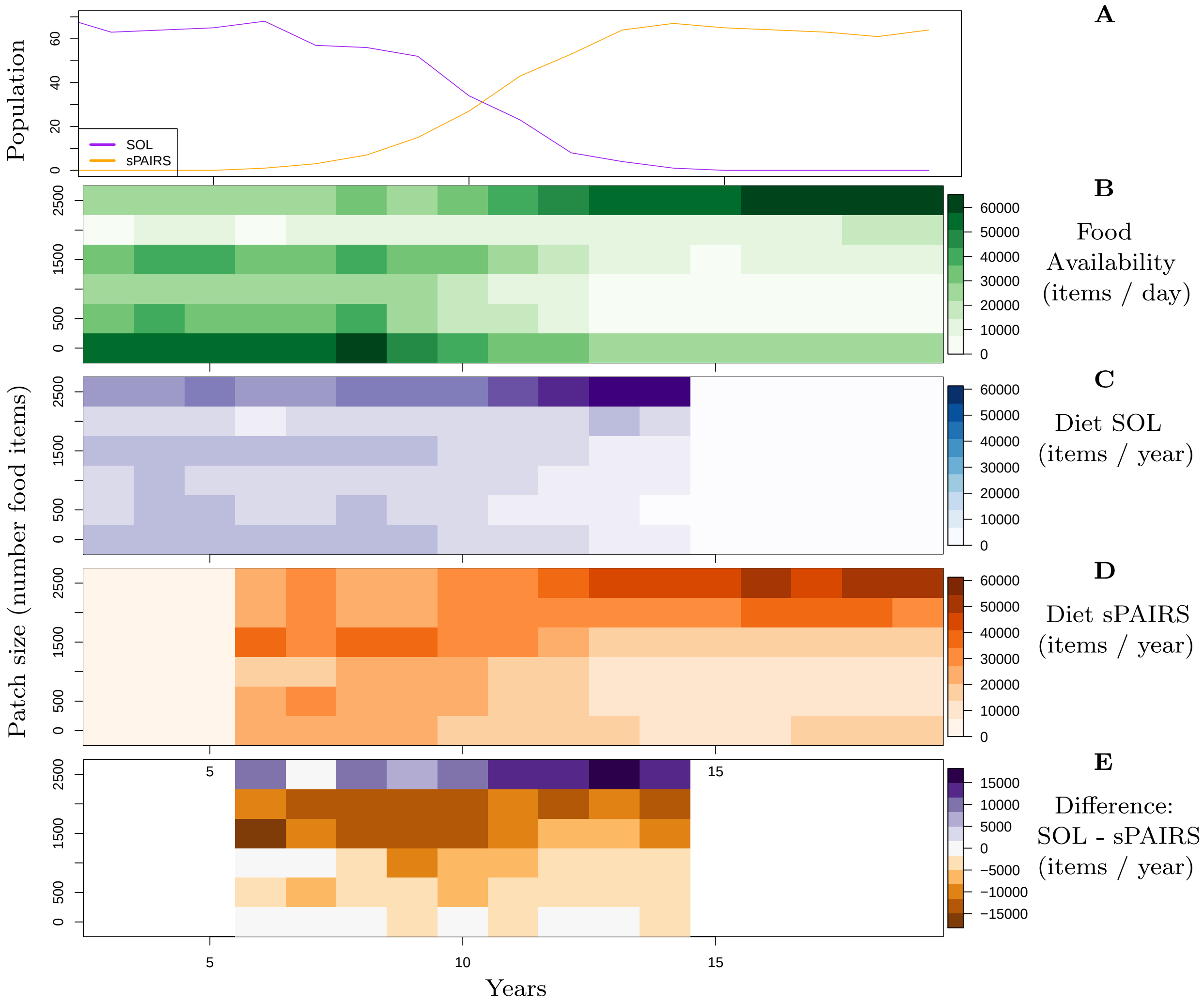

Figure S4: **Invasion of traveling pairs into solitary world: ecology and patch fragmentation niche**. (a) population size; (b) distribution of food in different patch sizes (fragmentation); (c) food intake of solitaries from different patch sizes; (d) food intake of groupies from different patch sizes; (e) difference food intake (solitary - groupies). sPAIRS: straight moving pairs. SOL: solitary individuals. Environment: Patch class III. Each grid square represents a year average.

7 Foraging success and assortment

The invasion simulations give insight into how grouping patterns determine fitness and natural selection. For invasion simulations we measured assortment s_i of genotypes into groups using the following measure:

$$s_i = \frac{g_{io}}{g_{iT}} - \frac{p_{io}}{p_{iT}} \quad (19)$$

where g_{io} and g_{iT} are the number of an individual's own type, and total number of individuals respectively, per group formed by individual i, and p_{io} and p_{iT} are the population of type i and the total population respectively at the moment the group formed. This measure reveals the deviation of the expected

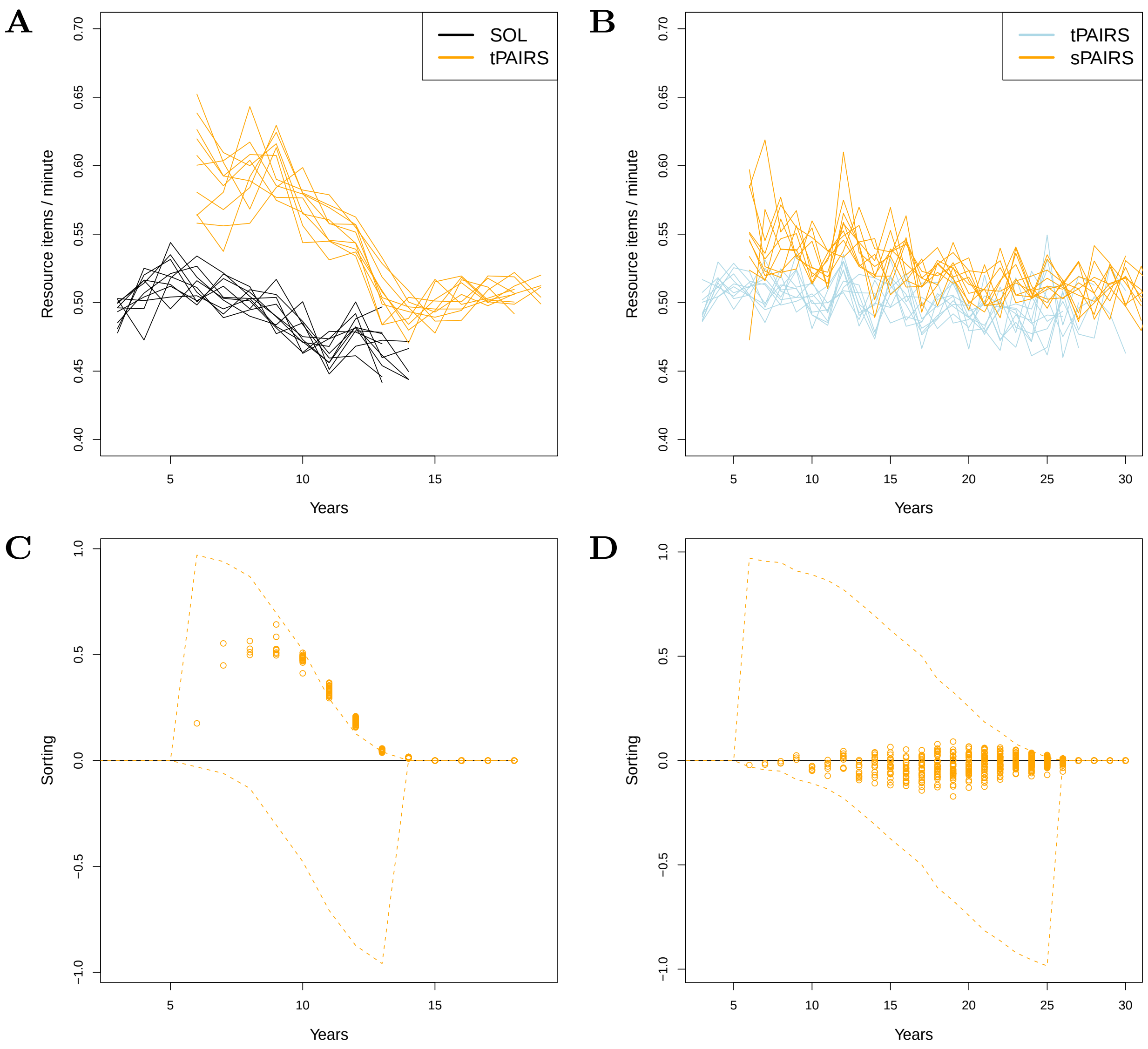

Figure S5: **Foraging efficiency and sorting**. (a,c) Straight travelling pairs (orange) invading population of solitaries (black), (b,d) Straight travelling pairs (orange) invading population of turning pairs with large turning angle (light blue). (a-b) foraging efficiency (averages of populations, 10 simulations), (c-d) sorting of invading type (fraction own type in groups - fraction own type in population). Dashed lines indicate max and min of sorting score for given population sizes. Data shown for all individuals of a single simulation (other simulations qualitatively similar). Patch class III.

proportions (i.e. due to population sizes) found in groups. Thus $s_i = 0$ indicates no assortment, and above and below 0 are positive and negative assortment. Note that the maximal assortment is a function of population size: for big populations positive assortment can never be a big deviation from no assortment and vice versa. With only one genotype there can be no assortment.

As shown, grouping individuals can invade and replace a population of solitary individuals (see Section 6). In fact, lone grouping individuals already have greater food intake rates than the average for solitary

individuals (Fig. S5a, start of orange lines greater than black lines). Such grouping individuals follow solitary individuals, and use the information solitary individuals provide about the location of food. Solitary individuals appear not to be directly affected by such grouping. One reason is that the grouping individual associates with many solitary individuals over time, diluting any disadvantages (or advantages) that solitary individuals may experience. Later, when more than one is present, grouping individuals group preferentially with each other, rather than with solitary individuals (positive assortment, Fig. S5c, orange circles).

However, whether grouping with a fellow grouping individual or a solitary individual, grouping individuals make use of "group-level" information processing about the location of food to enhance food intake: foraging success of grouping individuals depends on information acquired indirectly with another individual (a group-level process). Thus during the invasion of grouping individuals we see that there is a transition from individual-level to multi-level information processing (individual-level and group-level). The latter then becomes selected, making natural selection a multi-level process: foraging success is now based on group-level structures and its multi-level information processing consequences.

The transition from individual to multi-level information processing allows novel selection pressures to arise and group-level properties associated with enhanced patch depletion can become optimized. Such multi-level selection happens in the case of straight moving pairs (sPAIRS) invading a population of turning pairs (tPAIRS). Here selection acts on group-level turning: it selects those individuals that are more often in groups that travel straight, even though on the individual-level everyone is turning (i.e. "zig-zag") and group-level behavior is undefined. Unlike for grouping invading solitary individuals, here there is hardly any assortment between the two types of grouping (Fig. S5d). The lack of assortment is because tPAIR individuals are more strongly attracted to other individuals than sPAIR individuals due to a larger maximum attraction angle, and sPAIR-tPAIR groups last on average longer than sPAIR-sPAIR groups (results not shown). Positive assortment is therefore not necessary for selection of sPAIR individuals. To be selected it is sufficient that sPAIR individuals spend at least some time in straight moving groups, while tPAIR individuals cannot.

References

1. van der Post DJ, Semmann D: **Local orientation and the evolution of foraging: Changes in decision making can eliminate evolutionary trade-offs.** *PLoS Comput Biol* Accepted.
2. Couzin ID, Krause J, James R, Ruxton GD, Franks NR: **Collective memory and spatial sorting in animal groups.** *J Theor Biol* 2002, **218**:1–11.
3. Wood AJ, Ackland GJ: **Evolving the selfish herd: Emergence of distinct aggregating strategies in an individual-based model.** *Proc R Soc Lond B* 2007, **274**:1637–42.
4. Hogeweg P, Hesper B: **Individual-oriented modelling in ecology**. *Math Comput Model* 1990, **13**:83–90.
5. Takeuchi N, Hogeweg P: **Multilevel selection in models of prebiotic evolution II: a direct comparison of compartmentalization and spatial self-organization.** *PLoS Comput Biol* 2009, **5**:e1000542.
