# Supplementary material for: Patch depletion, niche structuring and the evolution of co-operative foraging
Source: BMC Evol Biol. 2011 Nov 17;11:335. doi: 10.1186/1471-2148-11-335 (PMC3306211; doi:10.1186/1471-2148-11-335)
Supplement: Additional file 2 — Movies in mini-website. The second file is Additional file2.zip, which is a ZIPPED folder "miniwebsite" with file index.html (HTML) in which GIF animations can be viewed. The GIF animations reside in subfolder "miniwebsite/movies". The html file (index.html) can be opened with any web-browser and shows links to three pages showing the GIF animations: (i) Animatic S1: Solitary foraging, (ii) Animatic S2: Traveling pairs foraging, and (iii) Animatic S3: Opportunistic grouping individuals that are foraging. Descriptions of the movies are given in "index.html". These animations should start playing as soon as the link to the page with the animation is opened using a web-browser. [file 1471-2148-11-335-S2.ZIP › miniwebsite/sol.html]

Animatic S1: Solitary foraging (SOL)

Additional file 2 of "Patch depletion, niche structuring and the evolution of cooperative foraging" by Daniel J van der Post & Dirk Semmann   
  
**Animatic S1: Solitary foraging (SOL)**
  
Solitary individual (SOL) foraging on a patch of food items. White: Individual; Black fading to dark green: trajectory of individual; Light green: food items; Dark green: background; This represents about 6 hours of foraging viewed on a 250 by 250 meter area.  
  

  
  
  
Click here to go Animations menu  
