# Supplementary material for: Patch depletion, niche structuring and the evolution of co-operative foraging
Source: BMC Evol Biol. 2011 Nov 17;11:335. doi: 10.1186/1471-2148-11-335 (PMC3306211; doi:10.1186/1471-2148-11-335)
Supplement: Additional file 2 — Movies in mini-website. The second file is Additional file2.zip, which is a ZIPPED folder "miniwebsite" with file index.html (HTML) in which GIF animations can be viewed. The GIF animations reside in subfolder "miniwebsite/movies". The html file (index.html) can be opened with any web-browser and shows links to three pages showing the GIF animations: (i) Animatic S1: Solitary foraging, (ii) Animatic S2: Traveling pairs foraging, and (iii) Animatic S3: Opportunistic grouping individuals that are foraging. Descriptions of the movies are given in "index.html". These animations should start playing as soon as the link to the page with the animation is opened using a web-browser. [file 1471-2148-11-335-S2.ZIP › miniwebsite/oppgr.html]

Animatic S3: Opportunistic grouping (OPP-GRa)

Additional file 2 of "Patch depletion, niche structuring and the evolution of cooperative foraging" by Daniel J van der Post & Dirk Semmann   
  
**Animatic S3: Opportunistic grouping (OPP-GRa)**
  
Opportunistic grouping (OPP-GRa) and foraging on patches. White: focal individuals (others black); Black fading to dark green: individual trajectory; Dark green: background; Light green: food items; Notice how individuals find the patch independently, then cooperatively deplete the patch and after leaving the patch split up. This represents about 11 hours of foraging on a 500 by 500 meter area.  
  

  
  
  
Click here to go Animations menu  
